# Hot Carrier Injection-Driven Nano-Interface Assembly for Hydrogen Generation

**DOI:** 10.1021/acsami.6c04250

**Published:** 2026-03-11

**Authors:** Jia-Zhen Zheng, Amit Kumar Sharma, Yen-Hsun Su

**Affiliations:** Department of Materials Science and Engineering, 34912 National Cheng Kung University, No. 1, Daxue Road, East District, Tainan City 701, Taiwan

**Keywords:** hot electron transfer, surface plasmon resonance, FDTD, generative reinforcement machine learning, FeVO_4_, photoelectrochemical cell, hydrogen generation

## Abstract

Harnessing hot electron transfer (HET) at plasmonic–semiconductor interfaces is a promising route to modulate charge carrier dynamics toward solar energy-driven water splitting for hydrogen generation. Popular semiconductor photocatalysts driving solar-to-hydrogen conversion, such as FeVO_4_, suffer from limited visible light absorption, electron–hole recombination, and aqueous instability, often seeking band-gap engineering or cation doping to improve their catalytic prowess. In this first-of-its-kind comprehensive study, we demonstrate a sequentially optimized procedure to obtain one-dimensional (1D) FeVO_4_ that is integrated with plasmonic nanoparticles (PNPs) to address these limitations. Anchored on the surface of the semiconductor, PNPs generate hot electrons upon visible light irradiation, that are then transferred to FeVO_4_. Finite-difference time-domain simulations verify the electromagnetic field distribution around the FeVO_4_–PNP. Additionally, Au, Au-urchin, Ag, and Au+Ag NPs were used to understand the effect of varying sizes, shapes, and plasmonic metals on the photocatalytic efficiency of FeVO_4_. Circularly polarized photon-triggered asymmetric hot carrier injection (from Au, Au-urchin, Au+Ag) and plasmon-induced resonance energy transfer (from Ag) reveal voltage-dependent interfacial dynamics that govern charge separation and hydrogen evolution efficiency. The experimental data was used to train a generative reinforcement learning (GRL)-based machine learning model to predict the optimum parameters for tunable band gaps and applied bias photon-to-current efficiency. This study thus lays the foundation for determining appropriate combinations of PNPs and other semiconductor materials for photoelectrochemical (PEC) applications.

## Graphical abstract

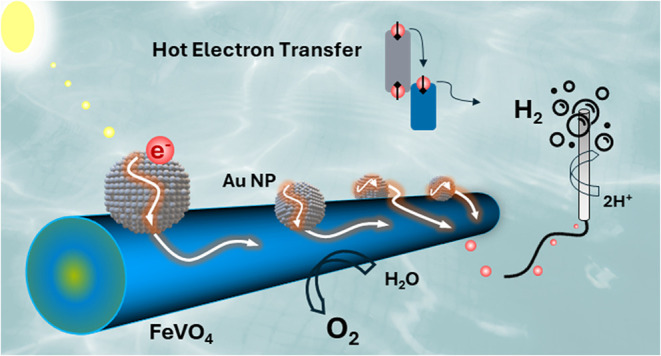

## Introduction

The recent decade has witnessed a plethora of semiconductor materials tailored to optimize light-harvesting efficiency and elevate the performance of next-generation photocatalysts toward clean hydrogen energy generation.^1^ The process of energy harvesting using sunlight—exploiting the incident photon energy—relies largely on the energy conversion efficiency of the semiconductor materials.^2^ The band-gap energy (*E*_BG_) of 2.0–2.2 eV is an established prerequisite for obtaining a measurable solar-to-hydrogen (STH) conversion efficiency in a photoelectrochemical (PEC) cell reaction.^3^ The incident photons with energy larger than *E*_BG_ successively excite the electrons from the valence band (VB) to the conduction band (CB) for charge migration.^4^ However, the separation and migration of electrons and holes as charge carriers is governed by the physical properties of the semiconductor.^5^

Metal vanadate semiconductors, such as FeVO_4_, have been strategically engineered into low-dimensional nanostructures such as nanorods, nanowires, and nanosheets,^5–9^ for precise *E*_BG_ modulation and enhanced charge transfer and to improve STH conversion efficiency. Previous studies have demonstrated that large specific surface area and Lewis basic sites in FeVO_4_ influence its photocatalytic activity^10^ for the oxygen evolution reaction,^11^ CO_2_ reduction,^9,12^ and energy storage.^13^ On the other hand, one-dimensional (1D) FeVO_4_·nH_2_O nanowires deliver a specific capacity of 1300 mAh g^–1^ for Li^+^ storage within the potential range of 0.02–3.5 V (vs Li^+^/Li), corresponding to approximately nine Li^+^ ions.^14^ Furthermore, a comparative analysis between 1D and bulk FeVO_4_ showed a better STH efficiency in the former due to their higher surface-to-volume ratio and enhanced carrier mobility.^15,16^

Heterostructure photoelectrodes, which are constructed by stacking two distinct semiconductors, have shown improved spatial separation of photogenerated charge carriers owing to the formation of an internal electric field at the interface, driving electrons and holes in opposite directions.^17^ Thus, energy band alignment between the two semiconductor layers critically influences the interfacial transport behavior. When the CB or VB offsets are too large, a significant energy barrier can be formed at the junction, suppressing photocurrent generation and ultimately impeding carrier migration.^18^

To enhance the charge transfer efficiency at the interface, hot carriers generated in plasmonic metal nanoparticles (PNPs) via localized surface plasmon resonance (LSPR) can be employed.^17,19–22^ LSPR is a phenomenon by which incident photon energy induces a collective oscillation of free CB electrons (intraband transitions) at room temperature.^5,23^ Additionally, the photon energy can trigger interband transitions, promoting electrons from the filled d-band to states above the Fermi level in the CB.^24^ The resonant excitation subsequently relaxes via a nonradiative and nonthermalized pathway within femtoseconds. This ultrashort dephasing process—mediated by Landau damping and acoustic phonon scattering—results in the generation of highly energetic nonequilibrium electron–hole pairs, known as “hot electrons”.^25–27^ The energy levels of these hot electrons or hot carriers do not conform to the conventional Fermi–Dirac distribution, while their energies are dictated by the energy of the incident photon.^28,29^ Hot electrons’ energy distribution is further attributed to the size of the PNPs owing to the corresponding changes in electron–phonon scattering within 100 fs.^29,30^ Notably, this ultrafast relaxation of hot electrons upon its interaction with the lattice electrons increases the lattice temperature.^31^ In a metal oxide semiconductor–PNP catalytic interface, such thermal effects are typically suppressed owing to a high Schottky barrier. A rapid extraction of these hot electrons, the most energetic and least scattered carriers, has immense potential to maximize the photocatalytic efficiency of hybrid catalysts. To further reduce recombination and enhance catalytic efficiency, this barrier can be engineered by a Schottky contact at the semiconductor–PNP interface.^32^

The efficiency of photocatalytic processes is critically dependent on light absorption, charge separation, charge migration, and prevention of charge recombination.^1^ Harnessing the LSPR characteristics of PNPs, such as gold,^11,33^ silver,^34,35^ and platinum^36^ nanostructures, would significantly resolve these shortcomings of metal oxide semiconductors.^37^ Previous reports show that gold NPs of variable sizes doped onto a metal oxide interface show 13.3% (5 nm NPs) to 3.3% (40 nm NPs) photon-to-electron conversion efficiency.^38^ The photogenerated carriers in Ag NPs-loaded FeVO_4_ composites could significantly enhance the photoreduction of CO_2_,^39^ while the SPR effect of Ag contributes to photon absorption and promotes electron charge transfer in a AgI-coated FeVO4/C3N4 plasmonic heterojunction.^40^ Ning et al. showed that the plasmon properties of Au- and Ag-decorated Fe_3_O_4_ composites could be regulated using polymers.^41^ TiO_2_ decorated with gold NPs has shown a 64-fold improvement in STH,^42^ while platinum nanoparticles on WO_3_ could yield a conversion efficiency of 7.6%.^36^ This is attributed to the LSPR-mediated hot electron transfer (HET), where the excited electrons are transferred from the PNPs to the semiconductor interface across the *E*_BG_.^43^ Additionally, plasmonic Ag NPs, upon absorbing incident visible light, can transfer the excited electron energy through dipole–dipole coupling, known as plasmon-induced resonance energy transfer (PIRET).^44^ HET and PIRET play a crucial role in modulating carrier dynamics at the semiconductor–PNP interface by generating a high density of localized electron–hole pairs at the surface.^45^ This phenomenon was reported in Au@Ag/AgI Schottky contact, leading to a significant improvement in the photocurrent response.^46^

At the interface of semiconductor–PNP catalysts, the incident photon triggers the electron–hole separation and generates hot electrons. These hot electrons are transferred to the semiconductor CB via HET, while the hot holes either recombine with the VB electrons or participate in the oxidation reaction. This recurring charge separation reduces electron–hole recombination and facilitates redox activity at the catalyst surface.^47^ Although this approach has been evaluated in detail, a systematic study assessing the impact of different PNPs on the photocatalytic efficiency of FeVO_4_ remains limited. This study addresses the gap by demonstrating that variations in the shape and size of PNPs could influence photon absorption and charge carrier dynamics at the interface.

Through sequential optimization, we demonstrate the synthesis of 1D FeVO_4_ and study its physicochemical characteristics. Subsequently, the ultraviolet (UV) reduction and self-assembly method was employed to embed colloidal Au, Au-urchin, colloidal Ag, and Au+Ag NPs onto FeVO_4_ to form a hybrid interface. Our results show that, in contrast to other combinations, FeVO_4_@Au exhibited excellent charge separation, electrode stability, and photocurrent density in a PEC cell for hydrogen generation. Additionally, finite-difference time-domain (FDTD) modeling simulation was used to compute the electrodynamic characteristics of the PNPs on the surface of FeVO_4_.^48,49^ Based on Maxwell’s equations, this numerical method assists in understanding the localization of photogenerated hot electrons at the interface of FeVO_4_.^50^

The influence of HET on PEC was investigated by irradiating spin-polarized photons onto the FeVO_4_@Au electrode. Spin selectivity excites electrons with defined spin orientation (spin↑ or spin↓), thereby injecting spin-polarized charge carriers into the semiconductor. An increased photocatalytic efficiency, evidenced by a higher hydrogen evolution rate, was compared with the other FeVO_4_@PNP electrodes. The experimentally obtained *E*_BG_ characteristics and applied bias photon-to-current (ABPE) efficiency of the hybrid catalyst were used to prepare a database for machine learning optimizations. This database can be used to expedite the process of selecting appropriate concentrations of the metal oxide and PNPs per the desired catalytic efficiency. We have used a generative reinforcement learning (GRL)^51^ model with a genetic algorithm neural network (GANN) to predict the optimum parameters governing the photocatalytic efficiency of the hybrid catalyst. GRL is a semisupervised machine learning model that allows human intervention^52^ to improve the accuracy of the predictions through a rigorous trial-and-error method.^53^ Through experimental results, the size of PNPs and their corresponding LSPR peak were identified as the major determinants of resultant photocatalytic efficiency through the HET mechanism. These factors were then selected as input parameters to train the GRL model optimizations by allowing the model to make decisions based on experimental ABPE. After successful training, the model was employed to predict *E*_BG_ and ABPE characteristics against variations in the size and LSPR peak of the PNPs.

## Results and Discussion

### Physical and Optical Characteristics of FeVO_4_

Scanning electron microscopy (SEM) images revealed the morphology and size of the FeVO_4_. Figure S1a,c,d predominantly shows the colloid morphology of FeVO_4_ obtained for samples A, C, and D, respectively, with an average particle size of 205 ± 25 nm (image processed using ImageJ) (Figure S1, see the Supporting Information). At a 0.1 mol/L precursor concentration (sample B), the resulting FeVO_4_ shows a one-dimensional growth with an average length of 1.42 μm and a height of 190.23 nm. This notable variation is attributed to the slower nucleation rate at lower concentrations, where the ion concentration in the solution is insufficient to support a high rate of uniform nucleation, resulting in the formation of uneven colloidal particles, as seen in sample A. At 0.1 mol/L, the anions in the precursor (nitrate in this study) preferably bind to one of the crystal facets and direct the growth of the colloid into a one-dimensional nanorod (sample B). After attaining a supersaturated state, the nucleation rate increases, leading to the formation of more uniform colloids of 92.71 and 273.02 nm (samples C and D, respectively). Transmission electron microscopy (TEM) images were obtained for these samples, as shown in Figure 1. High-resolution TEM images and neutron beam electron diffraction (NBED) reveal their corresponding lattice parameters and crystal planes. The *d*-spacings measured as 4.18, 5.59, 7.82, and 6.42 Å for samples A–D correspond to the (0–1–2), (011), (010), and (0–10) planes, respectively. NBED shows a highly symmetrical arrangement of diffraction spots, indicating good single-crystalline properties and long-range order.

**Figure 1:**
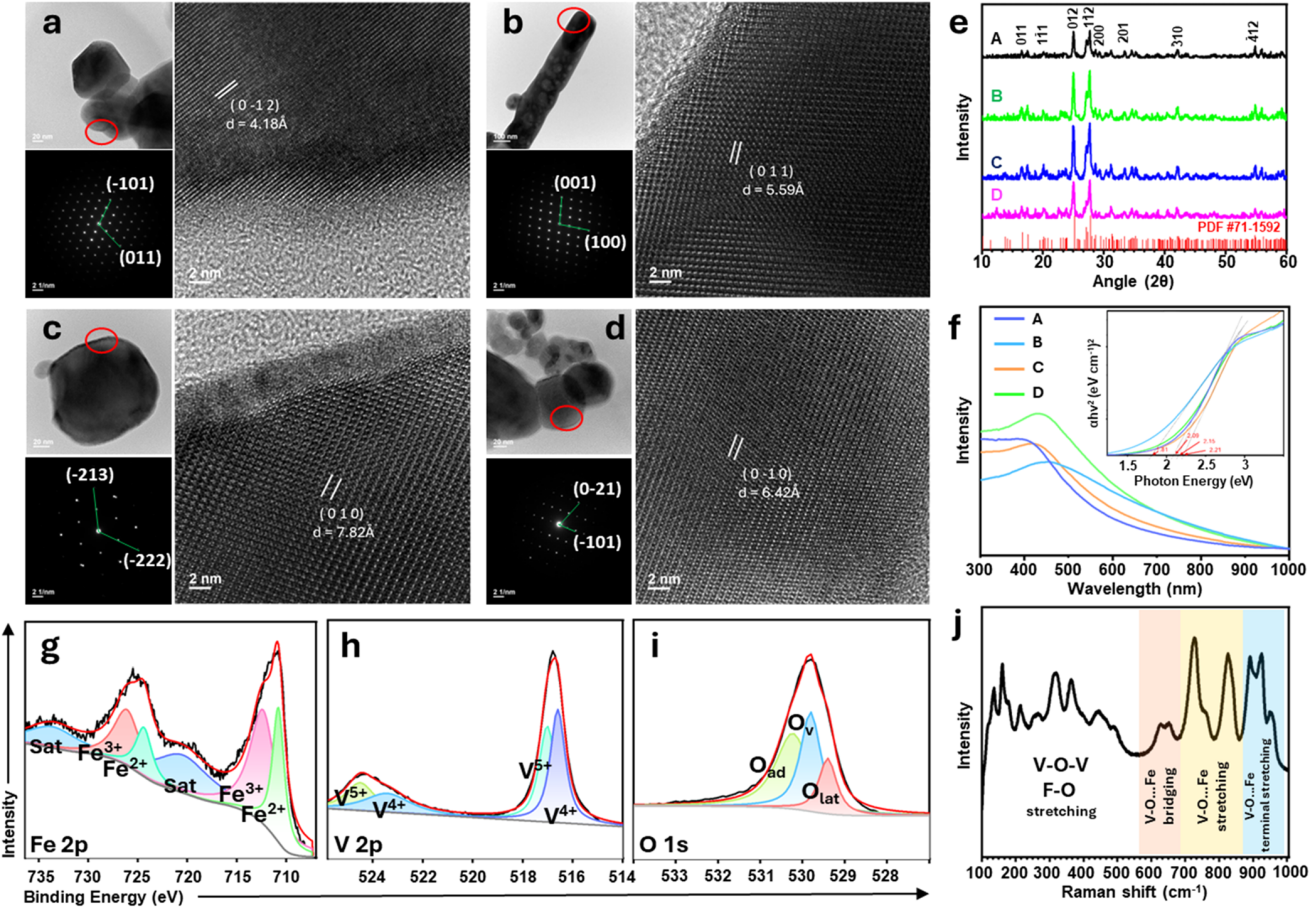
Physical and optical characterizations of FeVO_4_. Transmission electron micrographs, NBED, and HRTEM images of FeVO_4_ NPs prepared at (a) 0.05 mol/L for sample A; (b) 0.1 mol/L for sample B; (c) 0.2 mol/L for sample C; and (d) 0.3 mol/L for sample D. (e) XRD spectra of samples A, B, C, and D compared to the reference JCPDF #71–1592. (f) Absorbance spectra, inset: Tauc plot showing *E*_BG_ of samples A, B, C, and D. X-ray photoelectron spectra of (g) Fe 2p, (h) V 2p, and (i) O 1s show multivalent metals and oxygen-deficient surfaces in FeVO_4_. (j) Raman spectra of 1D FeVO_4_ (sample B) showing the inherent metal–oxygen bond vibrations.

X-ray diffraction (XRD) spectra revealed the crystal structure and defects in the as-synthesized FeVO_4_ (samples A, B, C, and D). The peaks apparent at 16.62°, 17.51°, 20.13°, 25.19°, 27.35°, 27.79°, 28.62°, 33.39°, 34.665°, 35.19°, 42.06°, and 54.8° correspond to (011), (−111), (1–11), (012), (−211), (1–12), (200), (201), (030), (2–21), (−310), and (−412) crystal planes, respectively. The characteristic peaks were consistent in all the samples, aligning well with the triclinic crystal system of FeVO_4_ (JCPDF #71–1592). Further, samples B and C exhibit better crystalline behavior that is attributed to the narrow fwhm of the characteristic peaks, indicating relatively suitable nucleation and growth rates. The optical absorbance of samples A, B, C, and D were used to calculate the *E*_BG_ of FeVO_4_ using the Tauc plot, as shown in Figure 1f. While all samples exhibit *E*_BG_ within the optimal range for photocatalytic activity, sample B exhibits the smallest *E*_BG_ (1.81 eV), attributed to its one-dimensional structure.^12,54^ A narrow band gap allows excited electrons to jump from the VB to the CB with less incident photon energy. Alternatively, the smaller *E*_BG_ further widens the range of visible light absorption up to the near-infrared regions. The irradiation with low-energy photons sufficiently generates a higher density of electron–hole pairs, which is suitable for PEC water splitting catalytic processes. Thus, sample B was chosen for subsequent experiments. The n-type semiconductor behavior of FeVO_4_ was confirmed using the Mott–Schottky (M–S) plot^55^ (Figure S2). The positive slope indicates that the primary charge carriers are electrons, and their concentration in the space charge layer increases with the applied bias potential. Notably, a smaller slope indicates higher carrier concentrations. Table S1 shows that sample B (slope 3.92 × 10^9^) has the highest carrier concentration (5.41 × 10^20^ cm^–3^) compared to other samples. This is consistent with the 1D structure of sample B, which facilitates efficient electron transport, a narrow band gap, and improved light absorption. The structural characteristics of 1D FeVO_4_ not only reduce the transition energy from the valence band to the conduction band but also enhance photogenerated carrier separation, making it suitable for photoelectrochemical reaction.

Figure 1g–i shows the high-resolution X-ray photoelectron spectra of Fe 2p, V 2p, and O 1s for 1D FeVO_4_ nanorods, showing multiple valence states of iron and vanadium. The peaks were assigned according to the NIST X-ray photoelectron spectroscopy database. Figure 1g shows the deconvoluted Fe 2p spectrum, revealing 2p_1/2_ states at 726.1 and 724.3 eV and 2p_3/2_ at 712.3 and 710.8 eV, attributed to Fe^3+^ and Fe^2+^, respectively. V 2p spectra, in Figure 1h, show 2p_1/2_ states at 524.5 and 523.4 eV and V 2p_3/2_ at 517.0 and 516.6 eV, attributed to V^5+^ and V^4+^, respectively. O 1s spectra reveal the O_L_ (lattice oxygen), O_v_ (oxygen vacancies), and O_ad_ (adsorbed oxygen) states at 529.4, 529.8, and 530.2 eV, respectively. O_ad_ is due to lattice oxygen, O_v_ is attributed to Fe–O–C, and O_ad_ is C–O derived from surface-absorbed oxygen. The oxygen vacancies can facilitate photocatalytic reactions in the sample.^16^ Furthermore, the nondegenerate vibrations in the triclinic phase were validated from the Raman shifts between 200 and 1000 cm^–1^ (Figure 1j). The lattice, translational, and vibrational bonds of the triclinic structure were observed between 100 and 400 cm^–1^. The stretching vibrations of Fe–O together with V–O bending modes and V–O–V bridging structures within the lattice appeared at 400 – 600 cm^–1^. The peaks at 650 cm^–1^ and 830 cm^–1^ indicate the characteristic stretching vibrations of V–O. The spectral region between 700 and 870 cm^–1^ shows the vibrational modes associated with V–O–Fe bridges, suggesting strong structural connectivity between vanadium and iron polyhedra. The higher wavenumber peak ascribed to terminal V–O stretching vibrations aligns well with previous reports.^13^ These characterizations confirm the synthesis of 1D FeVO_4_ with the desired electrical properties and surface chemistry.

### PNPs as Hot Electron Generators

To harness the photocatalytic activity of FeVO_4_, its visible light absorbance was engineered by decorating the pore cavities with PNPs. Four different PNPs, namely, gold nanoparticles (Au Nps), gold-urchin (Au-urchin), silver nanoparticles (Ag Nps), and gold + silver nanoparticles (Au+Ag Nps), were prepared. TEM images of colloids for Au, urchin-like protrusions at the edges of Au NPs, Ag NPs, and Au+Ag NPs, are shown in Figure 2a–d. The LSPR peaks reveal an absorption peak at 553 nm for Au NPs in aqueous solution; Au-urchin exhibits a broad absorption peak at 669 nm (Figure 2). Elemental analysis using energy dispersive X-ray spectroscopy (EDS) confirms the amount of metal in the PNPs (Figure S3). The uniform distribution of PNPs on the surface of FeVO_4_ was observed using SEM and EDS mapping (Figures S4 and S5). Figure S6 shows a detailed X-ray photoelectron spectroscopy (XPS) analysis of FeVO_4_@PNPs. Evidently, Fe 2p does not show significant peak shifts postintegration of PNPs. However, V^5+^ and V^4+^ states of FeVO_4_ tend to shift toward V^4+^ and V^3+^, while the O 1s spectra show changes in the O_v_ peak, with the highest for FeVO_4_@Au. The deconvoluted spectra of Au 4f and Ag 3d on the FeVO_4_@PNP samples did not reveal altered states of the PNPs, confirming that the PNPs exist in a metallic state without significant electron exchange with the semiconductor. The PNPs act as plasmon enhancers with minimal charge transfer binding interactions between the semiconductor and PNPs, and the sharp 4f_7/2_ and 4f_5/2_ peaks indicate band bending occurring primarily in the semiconductor, while the PNPs act as a Schottky contact (ultraviolet photoelectron spectroscopy, Figure S9).

**Figure 2:**
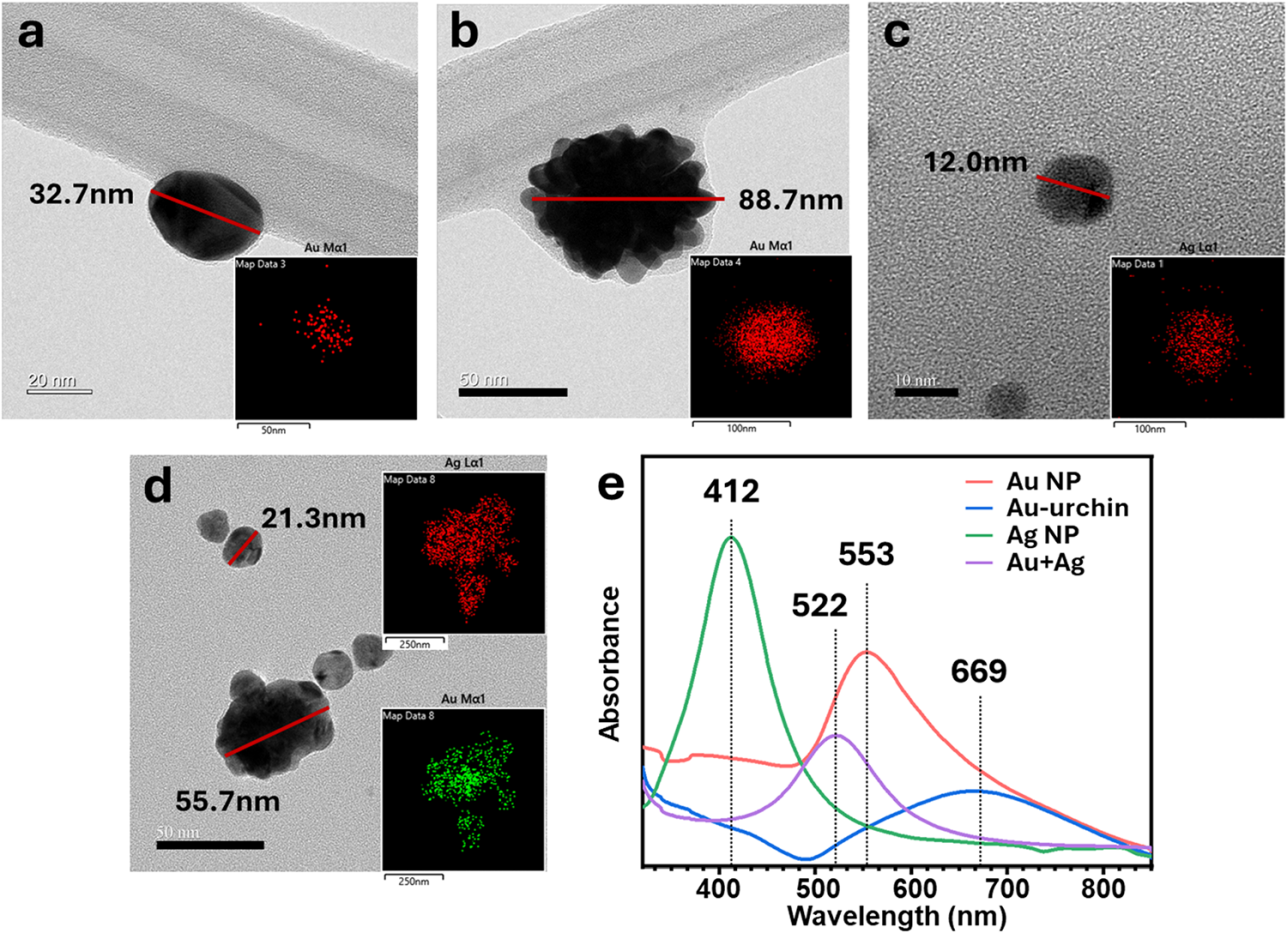
Physical and optical characterizations of PNPs. TEM representative images of (a) Au NPs; (b) Au-urchin NP; (c) Ag NPs; and (d) Au+Ag NPs, showing particle size and elemental distribution (inset). (e) UV–vis absorbance spectra of the PNPs, highlighting their respective absorbance maxima.

To elucidate the relationship of the PNP-induced scattered light on FeVO_4_, we employed FDTD to numerically simulate the LSPR characteristics of the PNPs. In accordance with Mie theory, for PNPs smaller than the wavelength of incident light, the interaction is dominated by the dipole approximation. The resonant oscillation of the induced dipole gives rise to the localized surface plasmon resonance (LSPR), which, in turn, leads to the scattering of the incident light. To assess this phenomenon, laser irradiation of wavelengths of 532 nm (Au), 658 nm (Au-urchin), 405 nm (Ag), and 532 nm (Au+Ag) was selected based on their maximum absorption peaks^56^ (Figure 2e). A semiconductor surface is constructed to show the 1D structure (shown in purple), while the PNPs are placed at its surface. The simulation yields an energy distribution contour plot that is measured using cT as the time step (shown in the lower panel of Figure 3). During the simulation, cT indicates the distance traveled by the incident photon, which is alternatively used to calculate the time (usually in femtoseconds) taken by the program to predict the electric field distribution. At the semiconductor interface, the electric field intensity from Au, Au-urchin, Ag, and Au+Ag varied significantly. It is seen that Au+Ag showed a relatively higher electric field distribution at cT = 2.495 μm, attributed to the synergistic effect, while Ag NPs showed a lower electric field distribution at cT = 1.97 μm. The maximum electric field is concentrated on one side of the spherical structures of Au and Au+Ag NPs and partially distributed around Au-urchin and in front of Ag NPs. This is attributed to the interaction between FeVO_4_ and the plasmon oscillation of the PNPs, congruent with the incident wavelength of light. The electric dipole oscillation and the direction of the incident light cross each other at the particle interface. Thus, upon interaction with FeVO_4_, the electrical dipole oscillates in the opposite direction of the electric field. The increase in the electric field distribution of Au+Ag NPs compared to Au NPs further indicates an enhancement in the local electromagnetic field interactions due to multiple dipole effects from the increase in particle size (∼50 nm). These results demonstrate the existence of hot spots, where the hot electrons from the PNPs are injected into the semiconductor FeVO_4_ surface during photocatalysis.

**Figure 3:**
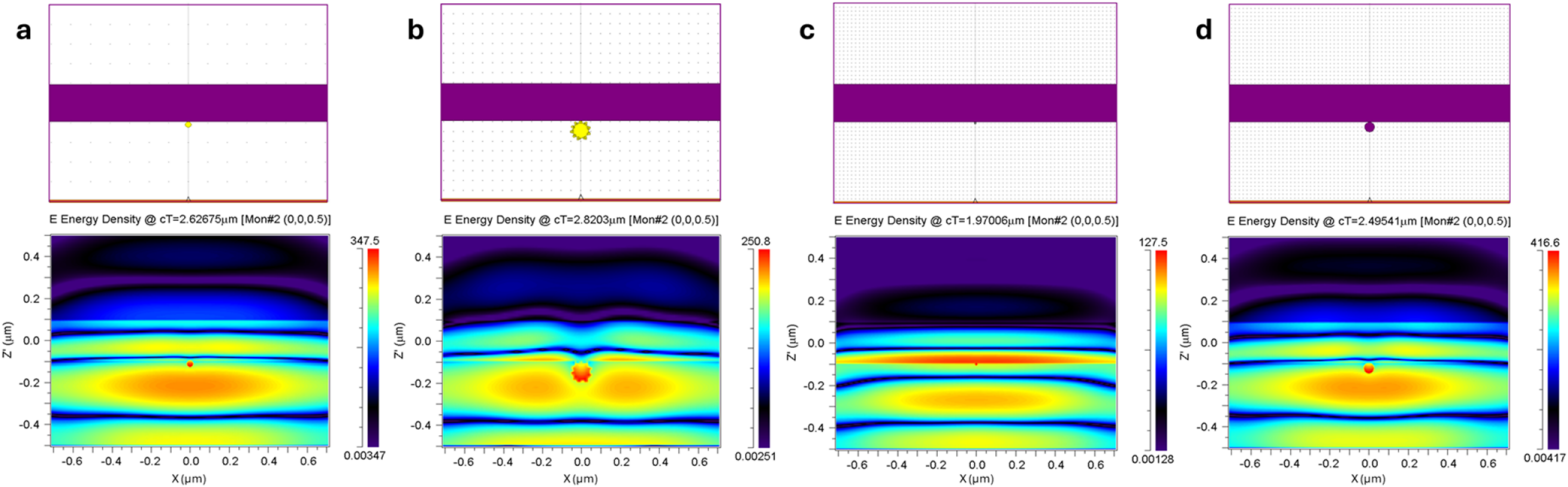
Finite-difference time-domain simulations. Upper panel: The purple-colored bar represents the 1D semiconductor surface, while the smaller yellow and purple spheres represent the PNPs. Lower panel: Energy density distribution after laser light interacts with the PNPs at the semiconductor–PNP interface. (a) FeVO_4_@Au NPs under 532 nm pulsed laser irradiation; (b) FeVO_4_@Au-urchin-like under 658 nm pulsed laser irradiation; (c) FeVO_4_@Ag NPs under 405 nm pulsed laser irradiation; and (d) FeVO_4_@Au+Ag NPs under 532 nm pulsed laser irradiation.

### Photocatalytic Performance of the FeVO_4_@PNP Photoelectrode

In order to verify the stable attachment of PNPs on the FeVO_4_ sample, SEM plane-view images and mapping analysis of the prepared photoelectrode were conducted. Figure 4a shows a representative SEM cross-sectional view of the photoelectrode with a uniform deposition of 1.36 μm prepared on an ITO substrate. Lateral SEM images and EDS analysis of the photoelectrode validate the distribution of PNPs on the semiconductor surface (Figures S4 and S5); the morphology of the PNPs is consistent with that of the TEM images. The hydrogen generation efficiencies of the photoelectrodes were evaluated using a linear sweep voltammetry (LSV) plot. A cyclic voltammogram was performed to activate the surface of the electrode before measuring the current density under illuminated conditions (Figure 4b) and dark conditions (Figure S7). A rapid increase in photoelectric current upon illumination was observed, while the measured onset potential was further lowered compared to the dark conditions. This shift indicates that the material requires lower energy to activate the redox reactions under bright light, which consequently improves the photoelectrochemical (PEC) efficiency.^57^ ABPE curve was then calculated using eq 2 (Figure 4c). The photoanodes of FeVO_4_, FeVO_4_@Au, FeVO_4_@Au-urchin, FeVO_4_@Ag, and FeVO_4_@Ag+Au showed maximum ABPE efficiencies of 0.14%, 0.27%, 0.072%, 0.17%, and 0.26% at 0.87, 0.78, 0.66, 0.82, and 0.77 V, respectively, with corresponding photocurrent densities of 0.26, 0.40, 0.08, 0.26, and 0.37 mA cm^–2^, respectively. FeVO_4_@Au achieved a 1.9-fold increase in ABPE%, which can be attributed to three reasons. First, the SPR nonradiative effect enhances current through HET and PIRET from PNPs to the semiconductor. The radiative effect of the PNP acts as a secondary light source, locally generating an electric field. Second, the combination of PNP and FeVO_4_ creates a heterojunction through the Schottky junction, as confirmed by the lattice pattern in the HRTEM image (Figure S8). The interplanar spacings of 0.23 and 0.26 nm correspond to the (111) and (110) crystal planes of Au NPs, respectively.^58^ A clear partial mosaic forms at the intersection of the semiconductor and Au NPs, which acts as the site for charge transport. Finally, the UV–vis spectrum (inset of Figure 2e) shows that the gold nanoparticles absorb in a wavelength range different from that of FeVO_4_, which increases the overall light absorption range of FeVO_4_, thus improving the ABPE efficiency.

**Figure 4:**
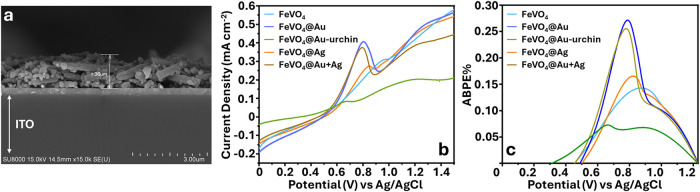
Electrochemical measurement of FeVO_4_@PNP photoelectrodes. (a) Representative cross-sectional view of the FeVO_4_@Au photoelectrode under SEM showing a uniform deposition of 1.36 μm on the ITO surface. (b) Linear sweep voltammetry plot. (c) Calculated ABPE% of FeVO_4_, FeVO_4_@Au, FeVO_4_@Au-urchin, FeVO_4_@Ag, and FeVO_4_@Ag+Au photoelectrodes.

### Working Mechanism of the FeVO_4_@Au Photoelectrode

To provide mechanistic insights into the photocatalytic activity of FeVO_4_@Au, the VB, CB, and work function (Φ) of the band structure were calculated based on the ultraviolet photoelectron spectra (UPS). The Fermi energy (*E*_F_) (3.06 eV vs vacuum) and secondary electron cutoff (*E*_SECO_) (16.73 eV vs vacuum) of FeVO_4_ and FeVO_4_@PNPs were obtained by extrapolating the intersection of the apparent tangent line and the line parallel to the baseline (as shown in Figure S9).^59^ Subsequently, the calculated energy gap between the CB of FeVO_4_ (−5.74 eV vs vacuum) and the HER potential (−4.44 eV vs vacuum) is a major hindrance to the charge transfer. The integration of PNPs with FeVO_4_ significantly modifies the electronic band structure of FeVO_4_@ Au, Au-urchin, Ag, and Au+Ag with *E*_F_ of 3.27, 4.25, 10.76, and 4.11 eV, and *E*_SECO_ of 17.25, 17.25, 19.16, and 17.51 eV, respectively. Consequently, the improved visible light absorption and charge transfer capabilities of the composite result in injection of SPR-mediated hot electrons into the semiconductor CB, pushing it closer to the HER potential.^18^ Furthermore, *E*_BG_ was derived from the Tauc plot. Upon exposure to visible light, the holes generated at the VB of FeVO_4_ can effectively participate in the oxygen evolution reaction (OER). However, the significant energy gap between the CB of FeVO_4_ and the hydrogen evolution reaction (HER) potential presents a challenge for efficient electron transfer, thereby limiting the overall ABPE%.

The incorporation of PNPs improves the band structure of the FeVO_4_@PNP heterojunction. The LSPR effect can concentrate light energy on the semiconductor surface while exciting the surface plasmons in the metal. These plasmons then transfer energy to the semiconductor. This process elevates the energy of the electrons in the CB of the semiconductor, thereby reducing the energy threshold needed for water splitting. Specifically, as shown in Scheme 1a, through the LSPR effect, the metal band aligns more closely with the HER potential, resulting in the coupling of the plasmons with the semiconductor’s band structure. This interaction enables the electrons in the semiconductor CB to gain more energy and effectively participate in the electron transfer necessary for the water splitting process. Thus, the introduction of PNPs not only enhances the light absorption efficiency of the semiconductor but also promotes the effective separation of electron–hole pairs by modulating its band structure.^39^ Combining UPS and optical analysis, these results reveal that the PNP modification not only optimizes the band structure of FeVO_4_ but also enhances carrier generation and transfer, providing a strong performance foundation for subsequent photohydrogen production systems.

**Scheme 1:**
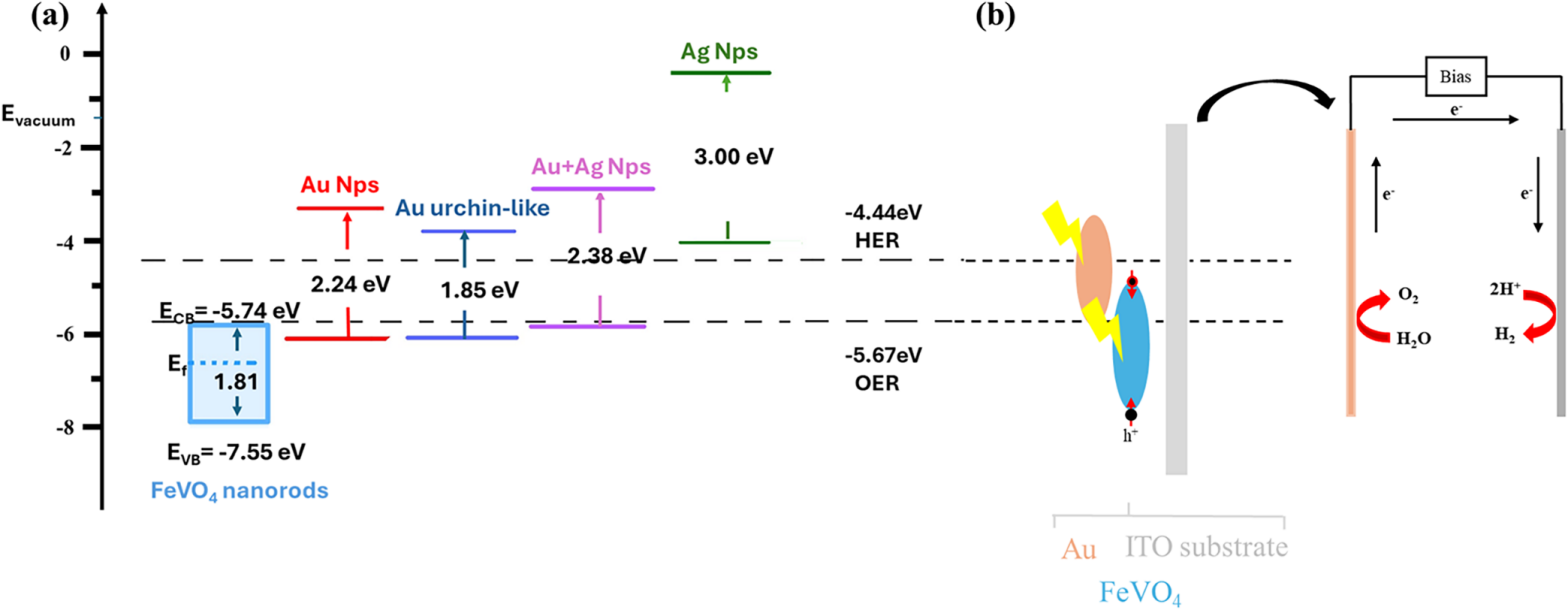
(a) Energy Band Diagram of FeVO_4_, Au NPs, Au Urchin-like, Au+Ag NPs, and Ag NPs. (b) Mechanism of Electron Transfer and Hydrogen Generation upon Applied Bias Voltage in a FeVO_4_@Au Photoelectrode Scheme

Scheme 1b sheds more light on the heterojunction of the photoelectrode. After the material absorbs visible light, the electrons in Au NPs, Au-urchin-like NPs, and Au+Ag NPs serve as light capture centers to induce plasmon resonance. The plasmon relaxation generates hot electrons and holes. The hot electrons overcome the Schottky barrier at the FeVO_4_/PNP interface and are injected into the FeVO_4_. Subsequently, the electrons traverse to the counter electrode (Pt) to induce the reduction reaction. In contrast, due to the significant energy band difference between Ag nanoparticles and FeVO_4_, electrons cannot be directly transferred to FeVO_4_. After Ag NPs absorb light, the electrons first migrate to the CB, return to VB, and release resonance energy that is utilized by FeVO_4_. This PIRET-triggered nonradiative energy transfers occur between plasmons and semiconductors sharing similar resonance energies.

### Effect of Spin Polarization on Charge Transfer

The charge transfer mechanism in FeVO_4_@PNP was further evaluated under spin polarization conditions, where, under light irradiation, electrons of a particular spin orientation are excited to the higher energy state for charge transfer, thus reducing the electron–hole recombination rate. An optical setup where the incident light from linearly polarized 405, 532, and 658 nm lasers were converted to a circular polarized (CP) light source using a quarter-wave plate. This CP light obeys ± ℏ angular momentum, which triggers spin-up (+ℏ) and spin-down (−ℏ) electronic excitations upon interaction with the semiconductor material.^60^ The angular momentum of the CP light can be controlled by changing the angle of the quarter-wave plate from 0° to 90°. This method was used to measure the subsequent current density from the photoelectrode under spin-up and spin-down conditions.

It is important to note that once the spin-controlled hot electrons are transferred to the conduction band of the semiconductor, they cannot revert to the PNPs due to the Schottky barrier.^61^ Notably, FeVO_4_@Au exhibited a higher current density in spin-down states, while the other FeVO_4_@PNPs showed relatively higher current density in spin-up states. The spin-controlled CP light could also trigger lower onset potentials in FeVO_4_@Au from 0.4 V (as shown in Figure 4b) to 0.08 V (as shown in Figure S10a). From Figure S10, the LSV plots reveal a higher current density for spin-down states for FeVO_4_@Au NPs, while for FeVO_4_@ Au-urchin and Au+Ag NPs, spin-up electron injections contribute to the higher current densities and reduced onset potentials. On the other hand, owing to the large difference in the energy bands between FeVO_4_ and Ag NPs, the electron transfer takes place through the formation of dipole coupling-mediated plasmon-induced resonance energy transfer between the semiconductor and the PNP. Furthermore, the polarization%, which is calculated using the LSV plot (using eq 1), refers to the degree of spin polarization of the current.

1 $$P=\frac{\left|J↑-J↓\right|}{J↑+J↓}$$

where *J*↑ is the current density due to spin-up electrons, and *J*↓ is the current density due to spin-down electrons. Particularly, it indicates the amount of current carried by electrons of spin-up (or spin-down) orientation compared with the total current. The % quantifies the magnitude of the current generated by spin-polarized electrons in a photocatalytic reaction. Figure S11 shows the plot of polarization% against the applied bias potential under irradiation with a specific laser. With 532 nm laser irradiation on FeVO_4_@Au photoelectrodes, the hot electrons from Au NPs originate at a very small bias potential of +0.05 V, while no significant polarization was observed between 0.1 to 1.2 V. This indicates that at a specific bias voltage, the energy alignment at the FeVO_4_@Au interface specifically allows a certain spin orientation (spin down) that are injected into the semiconductor. Similar observations were made for electrodes FeVO_4_@Au-urchin and FeVO_4_@Au+Ag at respective wavelengths, while FeVO_4_@Ag has peculiar behavior. At 405 nm spin-controlled laser exposure, a sharp peak at +0.19 V appears, followed by multiple broad peaks between +0.3 V and +0.4 V and between +0.88 V and +1.3 V, which is attributed to the spin-selective charge injection through dipole–dipole interaction between Ag NP and FeVO_4_. This corroborates that the spin-polarized electron injection into the semiconductor significantly enhances the electrochemical catalytic performance of the photoelectrode by reducing the charge recombination rate.

### GRL Model for Parameter Optimizations

The highly tunable size of PNPs influences the light absorption behavior of the hybrid photocatalyst, energy band structure, and, consequently, photocatalytic efficiency in conjunction with the semiconductor material. To accurately predict the fate of varied sizes of PNPs on the PEC activity, a GRL-based machine learning model incorporating GANN was tested on our experimental data. The size of the PNPs, absorption wavelength, and applied bias potential were used as training variables to predict the associated changes in the *E*_BG_ and ABPE% (Figure 5). The rigorous training pattern of the metaheuristic optimization GANN algorithm enables a subsequent improvement over the solutions for several generations. Through successive iterations, the algorithm generates predictions that are correlated with the experimental values. As shown in Figure 5, linear regression *R* ^2^ = 0.9942 validates the accuracy of the training model with minimal deviation from the input variables. The accuracy was determined based on the similarity between the experimental value and the predicted value.

**Figure 5:**
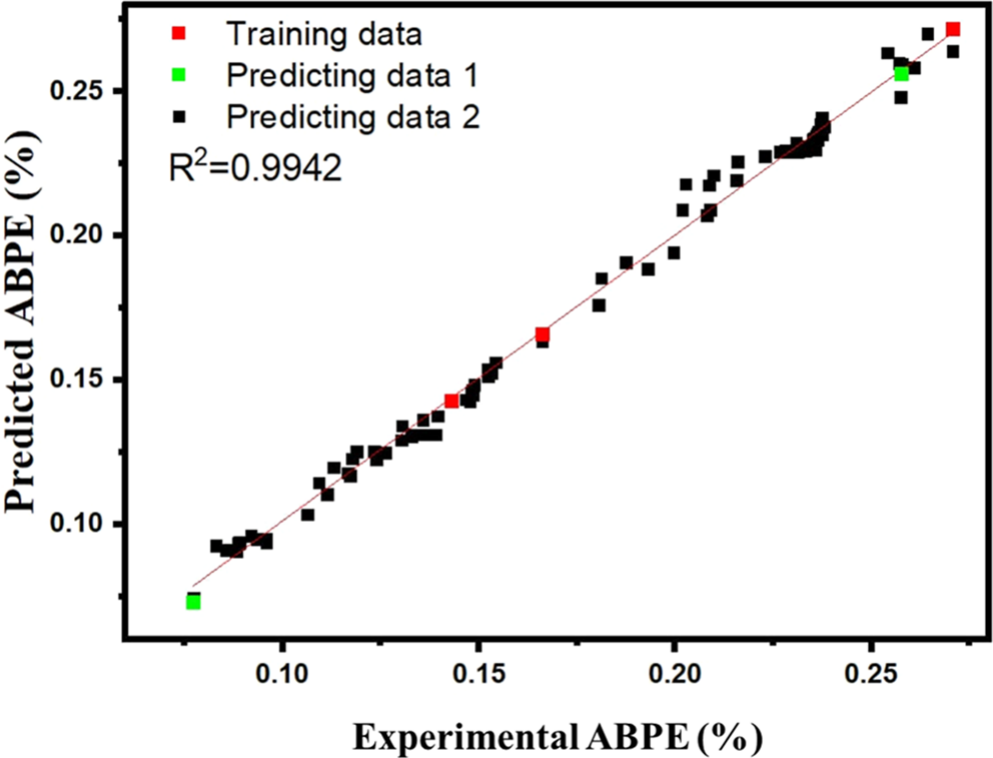
Training of the GRL model using the GANN algorithm. Regression analysis of the generative reinforcement learning (GRL) training and prediction model for FeVO_4_@PNPs.

The input parameters, namely, particle size, absorbance wavelength, and applied bias potential, were then selected with random sampling to obtain predictions *E*_BG_ and ABPE% (Table S3 and Figure 6). The results for FeVO_4_@Au show that a smaller Au particle size leads to a hypsochromic shift in absorption wavelength, resulting in wider *E*_BG_ and a decrease in ABPE% (Figure 6a). Similar trends were observed in other Au-PNPs; however, output predictions for Ag are contrasting. The increase in the particle size of Ag NPs tends to reduce *E*_BG_, consequently reaching relatively higher ABPE% at 0.75 V bias potential. This model can be used to further determine optimum parameters of the hybrid photocatalyst contributing to the PEC efficiency.

**Figure 6:**
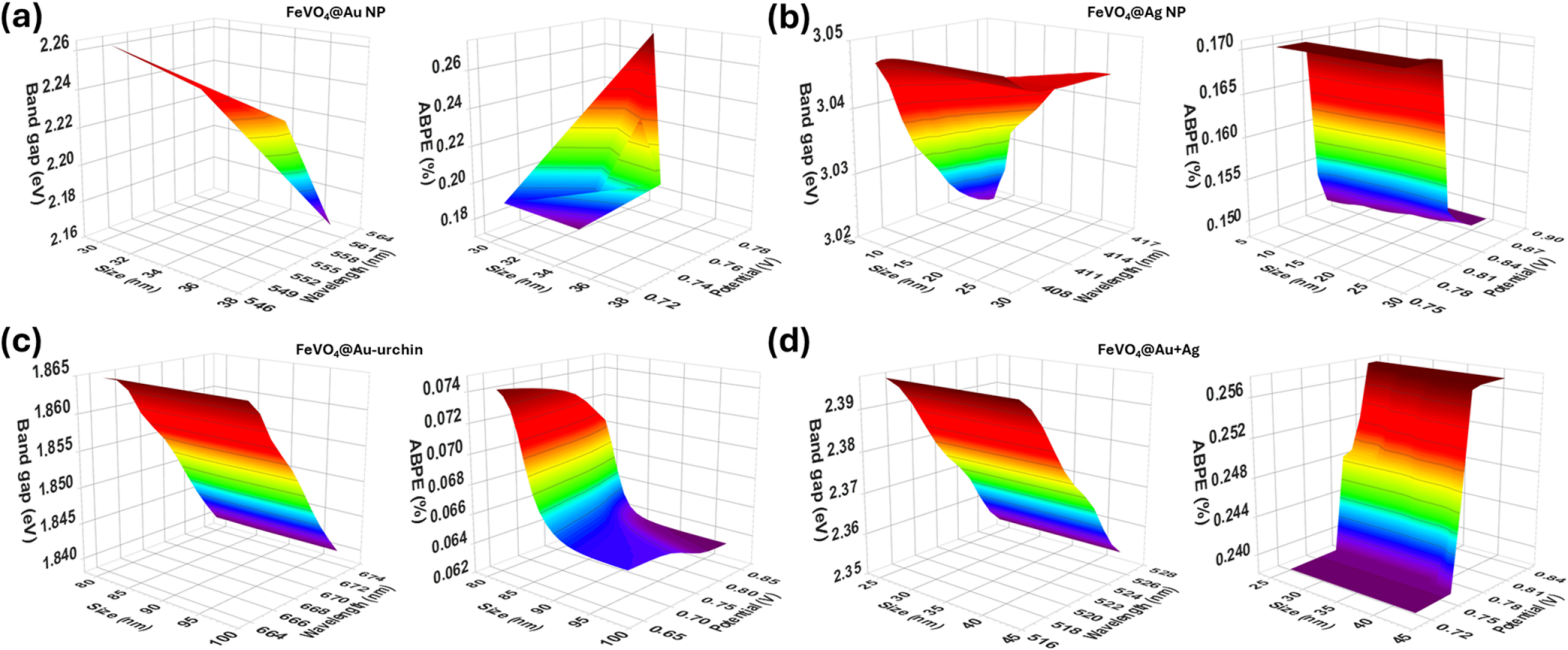
Output *E*_BG_ and ABPE% predictions by the GRL model. (a) FeVO_4_@Au; (b) FeVO_4_@Au-urchin; (c) FeVO_4_@Ag; and (d) FeVO_4_@Au+Ag.

### Stability of the Photoelectrode Post-Electrochemical Studies

The stability of the photoelectrodes post-electrochemical studies were also verified. SEM and EDS (Figures S12 and S13) analyses revealed the robust physical structure of FeVO_4_. A comparative analysis of Raman (Figure S14) and XRD (Figure S15) spectra before and after HER did not reveal any specific peak shifts in the FeVO_4_ crystal structure. The XPS spectra revealed oxidation of Fe 2p and V 2p after the HER experiments, which are attributed to the catalytic oxidation of the surface (Figure S16). Catalytic oxidation refers to the process in which the photoelectrode facilitates oxidation reactions during photocatalysis through water splitting. Consequently, minor, reversible shifts in metal oxidation states observed in the post-photocatalytic reaction are consistent with stable electrode behavior, where catalytic oxidation at the surface modulates the peak potential does not result in structural or chemical degradation. The minor, reversible shifts in the metal oxidation states are consistent with stable electrode behavior, as there is no evidence of structural disintegration or chemical degradation of the photoelectrode, as confirmed by Raman spectroscopy and XRD.

## Conclusion

This study uniquely presents a comprehensive investigation into the PEC activity of a PNP-modified FeVO_4_ photocatalyst. By systematically controlling the precursor concentration, 1D FeVO_4_ was successfully synthesized, exhibiting a narrow *E*_BG_, improved visible light absorption, and more effective charge carrier separation. The HET mechanism at the FeVO_4_/PNP interface was sequentially studied using FDTD simulations. FeVO_4_@Au nanorods achieved an exciting 1.9-fold increase in ABPE, suggesting that LSPR-induced HET, radiative electromagnetic field intensification, and optimized band alignment at the PNP/FeVO_4_ interface contribute cooperatively to the overall PEC performance of the photoelectrode. Band diagram analysis revealed that Au effectively modulates the energy landscape of FeVO_4_ by bridging the CB–HER potential gap. Mechanistically, HET was able to overcome the Schottky barrier, whereas Ag nanoparticles primarily contributed through radiative resonance energy transfer due to suboptimal band alignment. A GRL-based machine learning tool was used to predict optimum parameters for further alleviating *E*_BG_ and obtaining high ABPE%.

## Materials and Methods

### Materials

Ferric nitrate nonahydrate (Fe (NO_3_)_3_·9H_2_O), ethylene glycol (C_2_H_6_O_2_), sodium citrate dihydrate granular (HOC­(COONa)­(CH_2_COONa)_2_·2H_2_O), and potassium chloride (KCl) were purchased from J.T. Baker, the Netherlands. Ammonium vanadate (NH_4_VO_3_), 99%, and 1,6-hexanedithiol, 97% (C_6_H_14_O_2_), were obtained from Thermo Scientific. Hydrogen tetrachloroaurate­(III) trihydrate (HAuCl_4_·3H_2_O) was acquired from Alfa Aesar, and Sodium borohydride, 99% (NaBH_4_) was sourced from Aldrich. Potassium carbonate (KHCO_3_) was purchased from Showa Chemical Industry, Japan. 1-Hexanedithiol (C_6_H_12_S) was obtained from Macklin, and hydroquinone (C_6_H_4_(OH)_2_) was purchased from Toyama Chemical.

### Preparation of Different Morphologies of FeVO_4_

First, ammonium vanadate (NH_4_VO_3_) and ferric nitrate (Fe (NO_3_)_3_) precursors were prepared in a 1:1 molar ratio with different molar concentrations. 0.05 mol/L (sample A), 0.1 mol/L (sample B), 0.2 mol/L (sample C), and 0.3 mol/L (sample D) precursors were dissolved in DI water to obtain a homogeneous yellowish turbid solution. The mixture was then transferred to a stainless-steel autoclave lined with polytetrafluoroethylene (PTFE). Hydrothermal treatment was carried out at 180 °C for 3 h to allow the precursors to react. After cooling, an orange-brown precipitate was formed, which was filtered and washed multiple times with deionized water and anhydrous ethanol to remove any surface-bound impurities and contaminants. The resulting material was then vacuum-dried at 60 °C for 12 h to remove residual solvents. Following drying, the prepared sample was calcined in air at 550 °C with a heating rate of 5 °C·min^–1^ for 24 h to produce iron vanadate particles with distinct morphologies. The calcination process promotes the formation of the desired crystalline phase and morphology of FeVO_4_.

### Preparation of PNPs

Plasmonic nanoparticles were prepared as described in our previous literature,^62–64^ as follows.

#### Preparation of Au and Au-Urchin Nanoparticles

In this study, sodium citrate was employed as a reducing agent to synthesize gold nanoparticle (Au NP) seed solution via the reduction of chloroauric acid (HAuCl_4_) precursor salt. Initially, 50 mL of 0.5 mM HAuCl_4_ aqueous solution was prepared. Separately, 5 mL of 38.8 mM sodium citrate (Na_3_C_6_H_5_O_7_) solution was slowly added to the HAuCl_4_ solution under continuous stirring. The mixture was stirred with a magnetic stirrer and maintained at 100 °C with a stirring rate of 600 rpm for approximately 1 h. During the reaction, the solution gradually changed from colorless to ruby red, indicating the successful formation of colloidal gold nanoparticles. The final product was a colloidal suspension of Au NPs. To synthesize urchin-like gold nanoparticles, 30 μL of the previously prepared Au NP seed solution was added to 15 mL of 10–4 M HAuCl_4_ aqueous solution. After 2 min, 20 μL of 38.8 mM sodium citrate solution was slowly introduced as the reducing agent. Following thorough mixing, 2.5 mL of 30 mM hydroquinone (C_6_H_6_O_2_) aqueous solution was added, and the reaction mixture was stirred for an additional 30 min. A uniform colloidal solution of gold-urchin-like nanoparticles was then obtained and stored for further experiments.

#### Preparation of Au+Ag Nanoparticles

We prepared 15 mL of a 10^–4^ M chloroauric acid (HAuCl_4_) solution, 6 mL of a 10^–4^ M silver nitrate (AgNO_3_) solution, and 50 μL of a gold seed solution. Then, these three solutions were combined and stirred at room temperature for 5 min. Next, we added 20 μL of a 38.8 mM sodium citrate (C_6_H_5_Na_3_O_7_) solution, followed by 2.5 mL of a 30 mM hydroquinone (C_6_H_6_O_2_) aqueous solution. We maintained continuous magnetic stirring of the mixture for 30 min, which led to the formation of colloidal solution gold–silver alloy nanoparticles.

### Preparation of FeVO_4_@Au

An FeVO_4_ thin film was placed in a clean glass Petri dish, and 50 mL of 10^–4^ M chloroauric acid (HAuCl_4_) precursor solution was added to ensure complete coverage of the film surface. Subsequently, the Petri dish was transferred to a UV–ozone treatment, where ultraviolet-induced photoreduction was carried out for approximately 10 min to facilitate the reduction of Au^3+^ ions and the formation of gold nanoparticles (Au NPs) on the FeVO_4_ surface. Following the photoreduction process, the sample was removed and sequentially rinsed with deionized water and ethanol to eliminate unattached gold species and residual byproducts. The film was dried using nitrogen gas, and then an FeVO_4_@Au NPs thin film was obtained.

### Preparation of FeVO_4_@Ag, FeVO_4_@Au+Ag, and FeVO_4_@Au-Urchin

We performed the adsorption of colloidal silver, gold+silver alloy, and urchin-like gold nanoparticles onto the FeVO_4_ thin film via a self-assembly method, utilizing –SH functional group formation of robust Au–S and Ag–S bonds. First, we separately dissolved 0.5 g of 1-hexanedithiol (C_6_H_12_S) and 0.5 g of 1,6-hexanedithiol (C_6_H_14_O_2_) in 99 g of *n*-hexane (C_6_H_14_). We stirred the solution at 600 rpm for 15 min at room temperature to ensure complete homogenization. To enhance the adsorption capacity of the FeVO_4_ surface, we treated the thin film with UV–ozone for 30 min to remove surface carbon. Following surface activation, we immersed the substrate in the prepared solution and allowed it to react under static conditions for 24 h. Then, we rinsed the sample thoroughly on both sides with absolute ethanol to remove unreacted thiol molecules, followed by drying with nitrogen gas. The second UV–ozone treatment was conducted for 30 min. Then, the modified substrates were individually immersed in colloidal solutions of spherical silver, gold+silver alloy, and gold-urchin-like nanoparticles, allowing adsorption to proceed for 24 h. This step enabled self-assembly-driven deposition of PNP onto the FeVO_4_ surface via the –SH functional group. Upon completion of the adsorption process, we rinsed the samples with absolute ethanol to remove loosely bound nanoparticles and dried them using nitrogen gas. As a result, each type of PNP was stably immobilized on the FeVO_4_ thin film surface.

### Preparation of the FeVO_4_/PNP/ITO Electrode

The preparation process involves UV reduction and self-assembly methods. First, 50 mL of gold nanoparticle colloidal solution is poured into an FeVO_4_/ITO Petri dish, and reduction is carried out using UV ozone treatment. Other PNPs are incorporated through the self-assembly method. A mixture of 0.5 g of 1-hexanethiol, 0.5 g of 1,6-hexanedithiol, and 99 g of *n*-hexane is prepared, and the FeVO_4_/ITO is immersed in this solution for 24 h to facilitate the formation of sulfhydryl group (–SH) bonding. The sample is then dried with nitrogen gas, followed by immersion in a PNP solution, where it is left undisturbed for 1 day. Finally, the sample is dried again using nitrogen gas to obtain a uniform FeVO_4_@PNPs electrode.

### Electrochemical Experiments and Hydrogen Generation

Photoelectrochemical measurements were performed using a CH Instruments (CHI) setup under visible light irradiation from an AM_1.5G_ solar simulator. Current–voltage (*J*–V) analysis was conducted for the FeVO_4_ and FeVO_4_@PNPs photoelectrodes. The photoelectrodes were used as working electrodes, Ag/AgCl was used as a reference electrode, and Pt was used as a counter electrode in a 1.0 M KHCO_3_ electrolyte. The applied potential range for the measurements was from 0 to 1.5 V, and the scan rate was set to 0.1 V/s. The applied bias and photoelectric conversion efficiency were calculated using the ABPE method. To evaluate photon-to-current efficiency, the ABPE% (applied bias photon-to-current efficiency) was calculated using eq 2

2 $$\mathrm{ABPE}\;\left(\%\right)=\left(\frac{J_{\mathrm{ph}}\times \left(V_{\mathrm{app}}-V_{0}\right)}{P_{\mathrm{in}}}\right)\times 100$$

where *J*_ph_ is the photocurrent density (mA/cm^2^), *V*_app_ is the applied bias voltage (V), *V*_0_ is the theoretical water splitting voltage, typically 1.23 V, and *P*_in_ is the incident light power density (W/cm^2^).

### Finite-Difference Time-Domain Simulations

To simulate the optical properties of FeVO_4_@PNP, we employed commercial FullWave FDTD Simulation Software with RSoft CAD, Synopsys, CA. The surface morphology of the PNP-decorated FeVO_4_ photoelectrodes, as observed by SEM, was used as the basis for constructing the FDTD simulation models. To investigate the influence of different PNPs on electromagnetic field enhancement, four distinct models were established. Corresponding to the absorption peak wavelengths of the respective nanoparticles, pulsed laser sources were set at 532, 658, 405, and 532 nm for Au, Au-urchin, Ag, and Au+Ag NPs, respectively. These wavelengths were used as the incident light source to analyze the resulting spatial distribution of the electric field energy density upon illumination. A total-field scattering field (TFSF) source was employed to separate the incident and scattered fields, while perfectly matched layers (PMLs) were applied in all spatial directions to absorb outgoing waves. A nonuniform mesh was used in the simulations, with a minimum mesh step of 0.25 nm. A linearly polarized light source was considered, with its polarization direction aligned along the longitudinal axis of the nanorods.

### Computational Details of the GRL Model

Experimentally obtained *E*_BG_ and ABPE% for FeVO_4_, FeVO_4_@Au, and FeVO_4_@Ag were used to train the generative reinforcement learning (GRL) model. A generative artificial neural network (GANN) was used to make preliminary predictions.^65^ SuperPC Neuron 5.0 was used to perform the machine learning experiments. A regression analysis of the predicted ABPE% (FeVO_4_@Au-urchin and FeVO_4_@Au+Ag) against the experimental ABPE% was performed to determine the accuracy of the training model. The results were compared with the experimentally obtained values of *E*_BG_ and ABPE%. Particle size (nm), wavelength (nm), and applied bias potential (V) were used as input variables, while *E*_BG_ and ABPE% were used as output variables. The output data was repeatedly trained so that the accuracy of the model was high with minimal deviations against the input data. About 2000 data points were then randomly selected, representing the size of the PNPs, the wavelength of light, and an ABPE% with similar FeVO_4_ parameters, and introduced to the training set to accurately predict the consequential change in ABPE% and *E*_BG_ in a larger data set.

## Supplementary Material



## Notes

SEM and EDS analysis of FeVO_4_, FeVO_4_@PNPs, and photoelectrodes; electrical characterizations such as Mott–Schottky plot; cyclic voltammogram; linear sweep voltammograms; chronoamperometry scan of photoelectrodes; structural characterizations such as XPS, UPS, XRD, and Raman spectroscopy of FeVO_4_ and FeVO_4_@PNPs; and factors used for machine learning experiments (PDF)
