# Supplementary material for: Hot Carrier Injection-Driven Nano-Interface Assembly for Hydrogen Generation
Source: ACS Appl Mater Interfaces. 2026 Mar 11;18(11):16627–38. doi: 10.1021/acsami.6c04250 (PMC13022809; doi:10.1021/acsami.6c04250)
Supplement: Supplementary file 1 [file am6c04250_si_001.pdf]

Supporting Information

Hot Carrier Injection Driven Nano-Interface Assembly for Hydrogen Generation

*Jia-Zhen Zheng†, Amit Kumar Sharma†, Yen-Hsun Su**

Department of Materials Science and Engineering, National Cheng Kung University, Tainan City 701, Taiwan

† These authors have contributed equally to this work.

*Corresponding Author: Yen-Hsun Su (yhsu@mail.ncku.edu.tw)

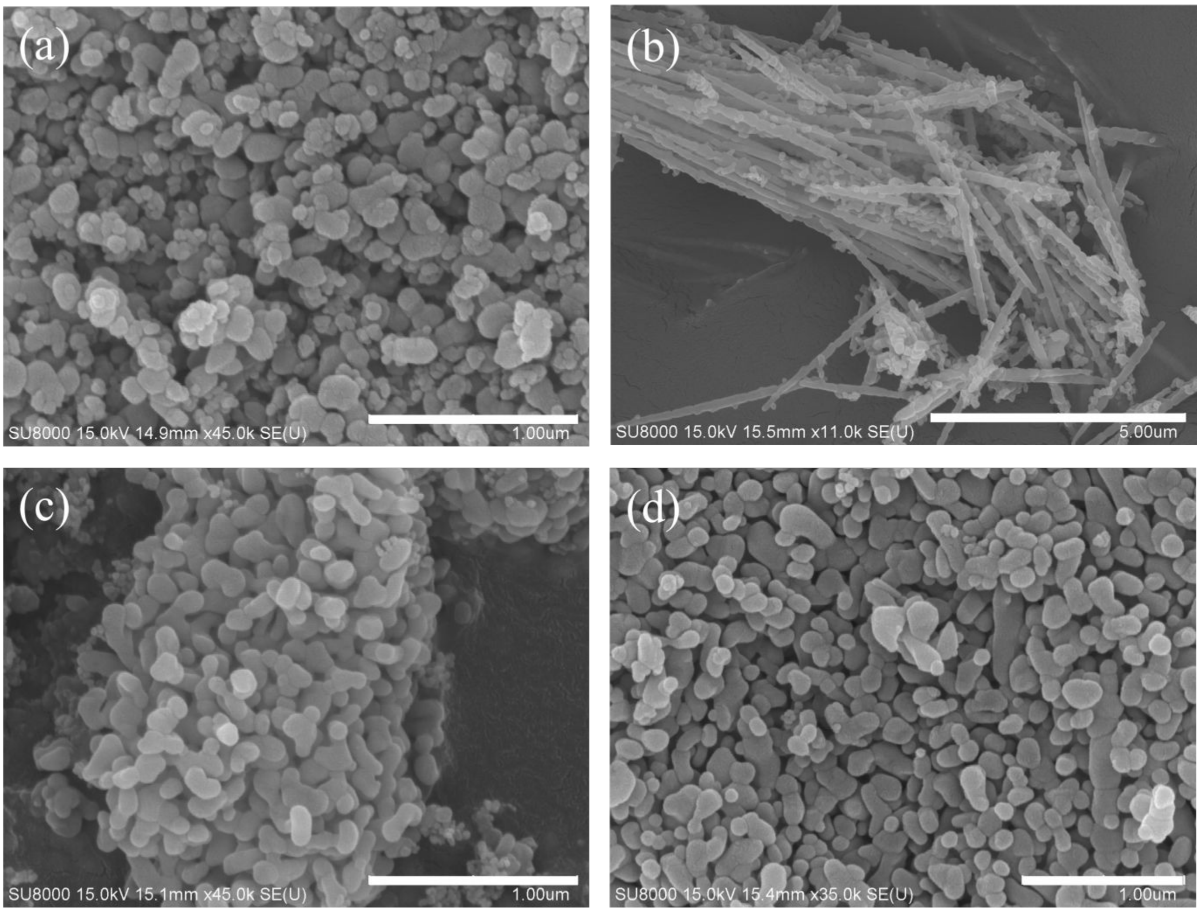

Figure S1. Scanning Electron Micrographs of $FeVO_4$ NPs prepared at varying precursor ratios. (a) colloidal particles obtained at 0.05 mol/L, Sample A; (b) one-dimensional nanostructure obtained at 0.1 mol/L, Sample B; (c) colloidal particles at 0.2 mol/L, Sample C; (d) larger colloids at 0.3 mol/L, Sample D.

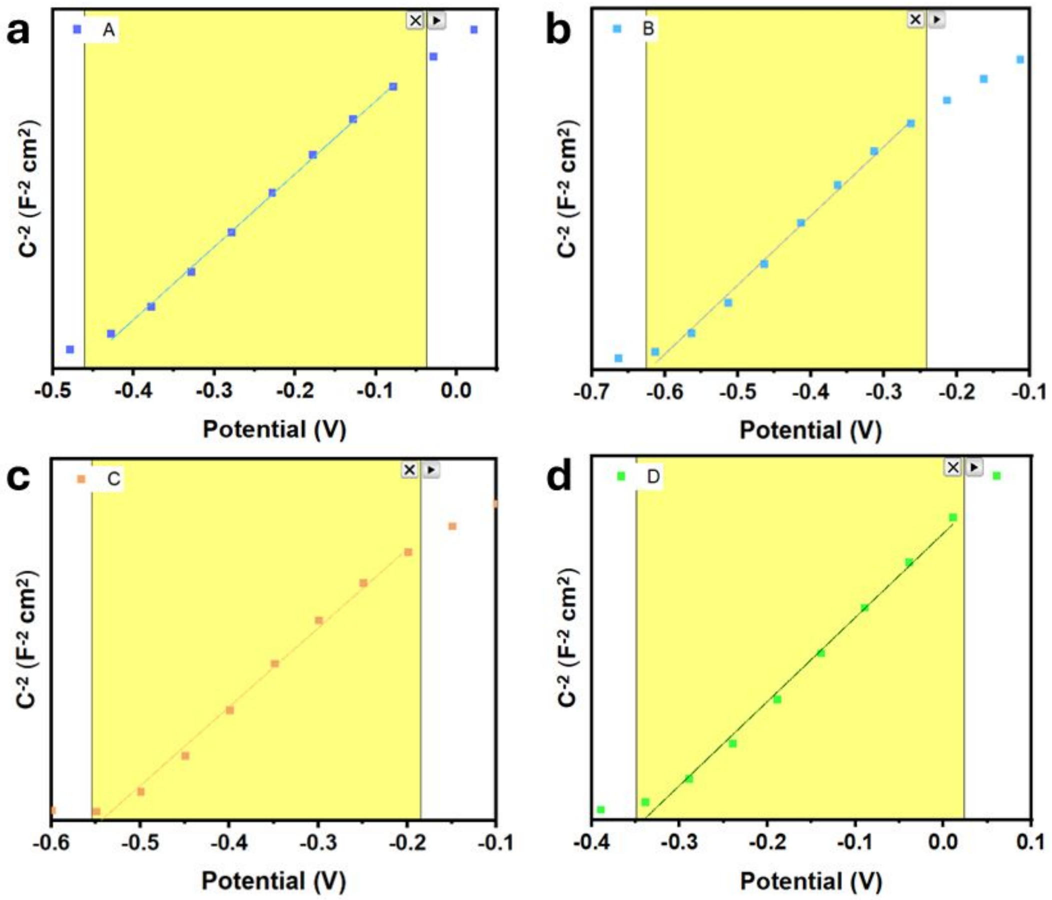

Figure S2. Mott-Schottky (M-S) plot of $FeVO_4$ prepared at (a) 0.05 mol/L, Sample A; (b) 0.1 mol/L, Sample B; (c) 0.2 mol/L, Sample C; (d) 0.3 mol/L, Sample D.

M-S measurements were performed using a three-electrode system at 1000 Hz. The linear relationship between $1/C^2$ and the applied voltage is expressed as Equation 1 [1, 2]:

$$\frac{1}{C^2} = \frac{-2}{eN_d A^2 \varepsilon\varepsilon_0}\left(V - V_{fb} + \frac{kT}{e}\right) \;\ldots\ldots\; (1)$$

Where C is the interfacial capacitance, e is elementary charge, ε is dielectric constant, ε_0 is the permittivity in vacuum, N_d is the carrier charge concentration, V is the applied bias potential, V_{fb} is the flat band potential, k is the Boltzmann constant, T is the temperature.

The carrier charge concentration was calculated using Equation 2.

$N_d = \frac{2}{slope \times e\varepsilon\varepsilon_0}$ …….. (2)

Table S1: Carrier concentration calculated from the M-S plot

	Sample A	Sample B	Sample C	Sample D
**Slope (×10^9)**	4.67	3.92	5.42	5.62
**N_d (×10^{20}) (cm^{-3})**	4.42	5.41	3.77	2.79

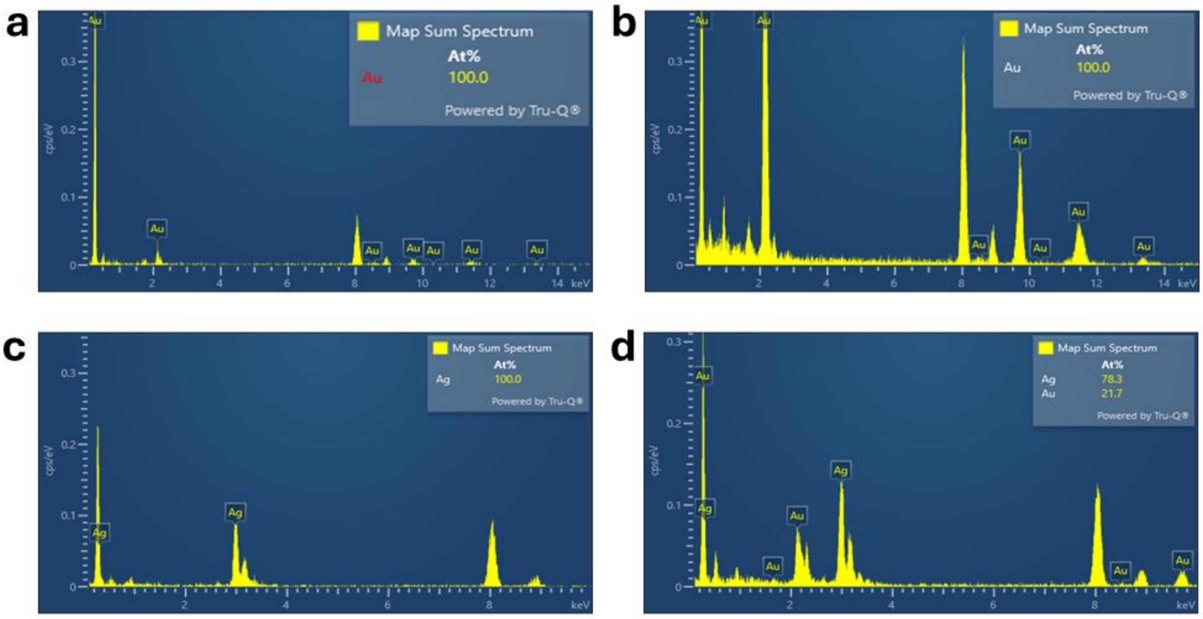

Figure S3. Energy dispersive X-ray spectroscopy of (a) Au NPs, (b) Au-urchin, (c) Ag NPs, and (d) Au+Ag NPs.

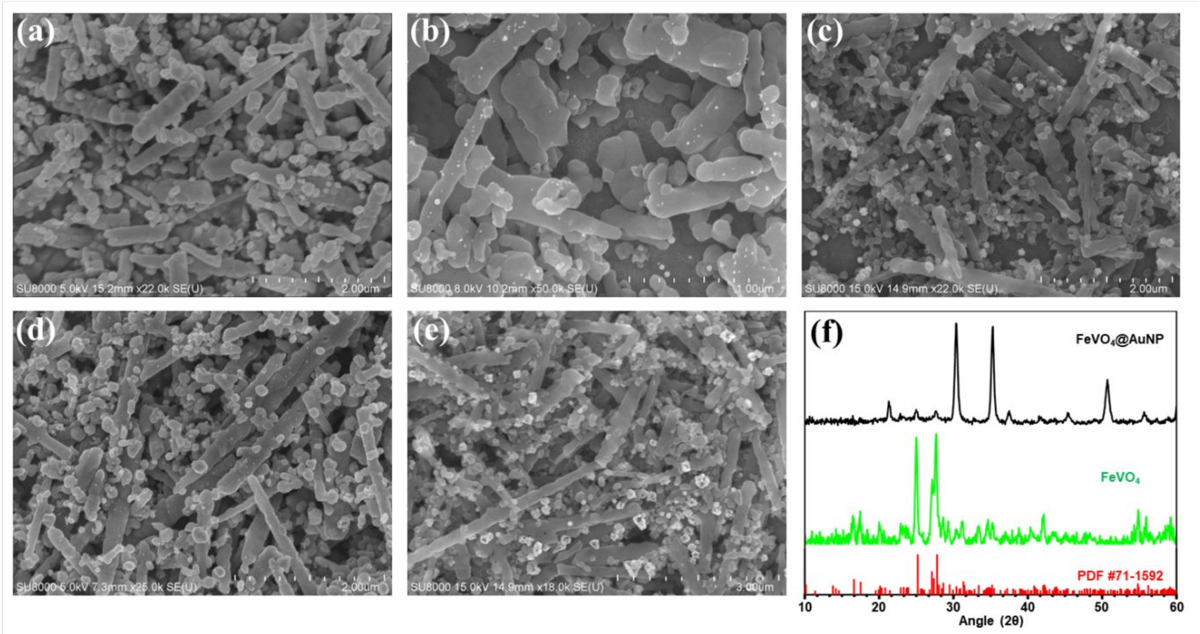

Figure S4. SEM images of (a) $FeVO_4$, (b) $FeVO_4$@Au, (c) $FeVO_4$@Au-urchin, (d) $FeVO_4$@Ag, and (e) $FeVO_4$@Ag+Au photoelectrode. (f) XRD spectra comparing planes in $FeVO_4$ and $FeVO_4$@AuNP.

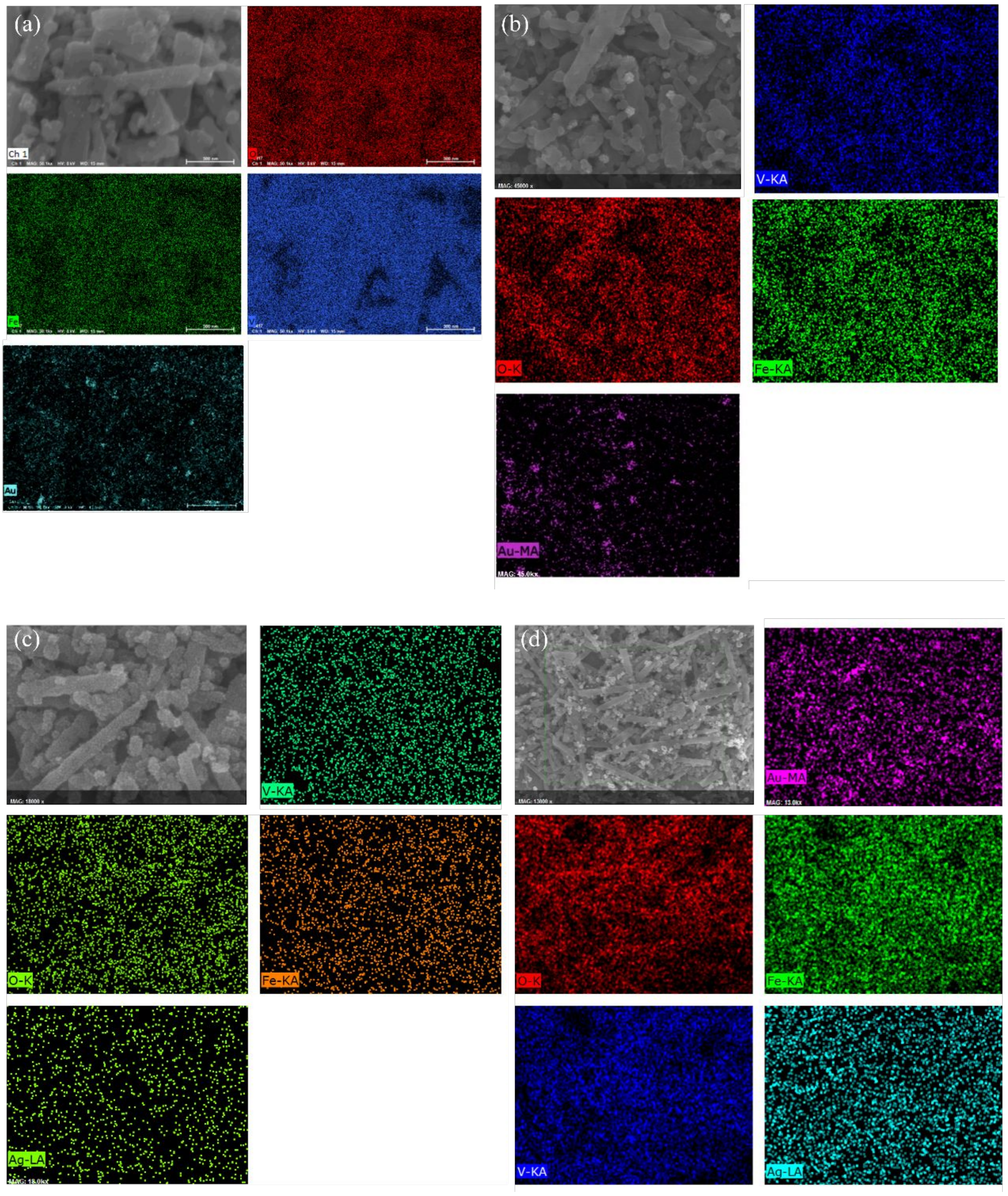

Figure S5. SEM mapping of elemental distribution of $FeVO_4$@ (a) Au NPs, (b) Au urchin-like, (c) Ag NPs, and (d) Au+Ag NPs.

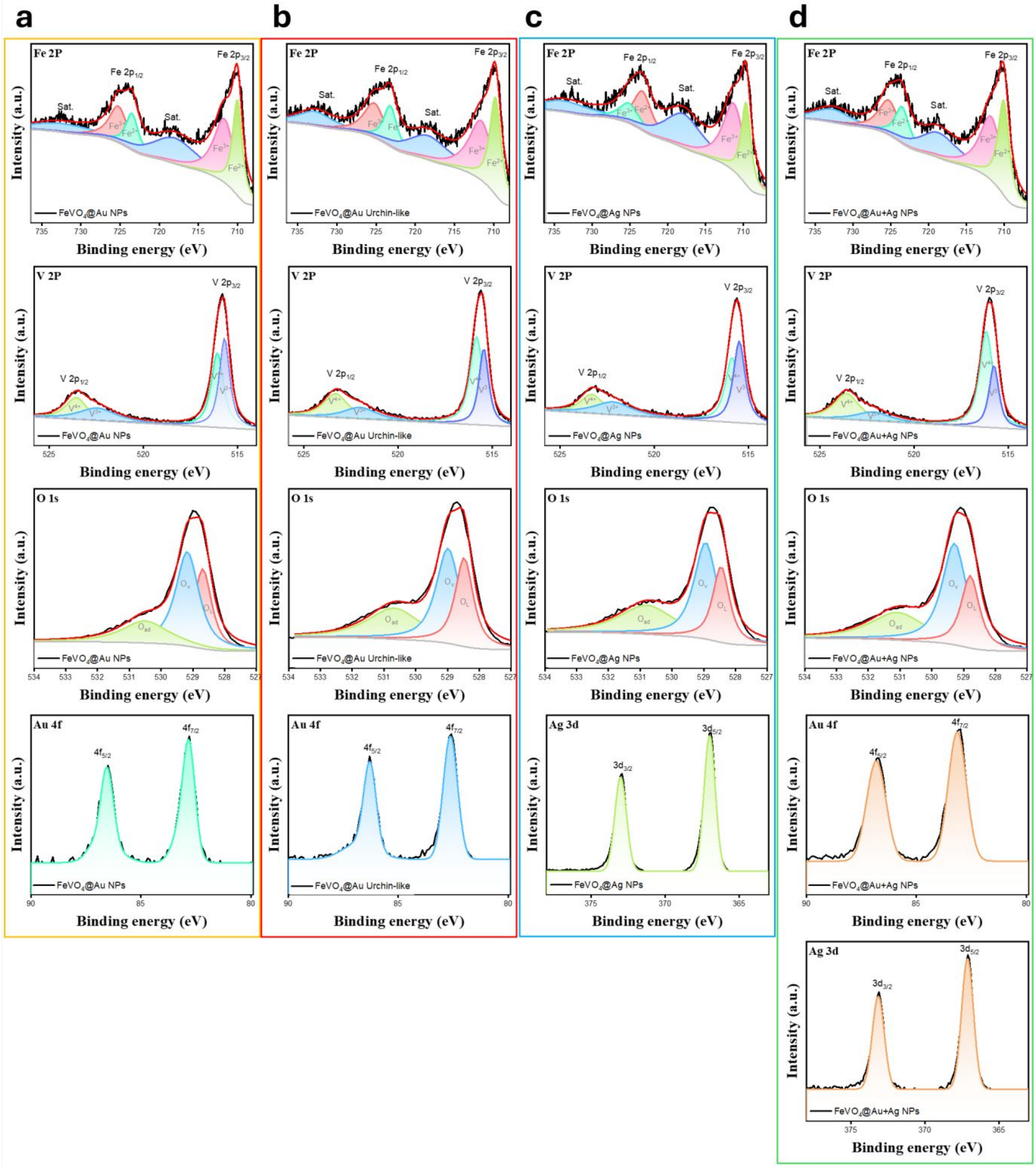

Figure S6. XPS spectra of (a) Au 4f on $FeVO_4$@Au; (b) Au 4f on $FeVO_4$@Au-urchin; (c) Ag 3d on $FeVO_4$@Ag; (d) Au 4f on $FeVO_4$@Au+Ag; (e) Ag 3d on $FeVO_4$@Au+Ag.

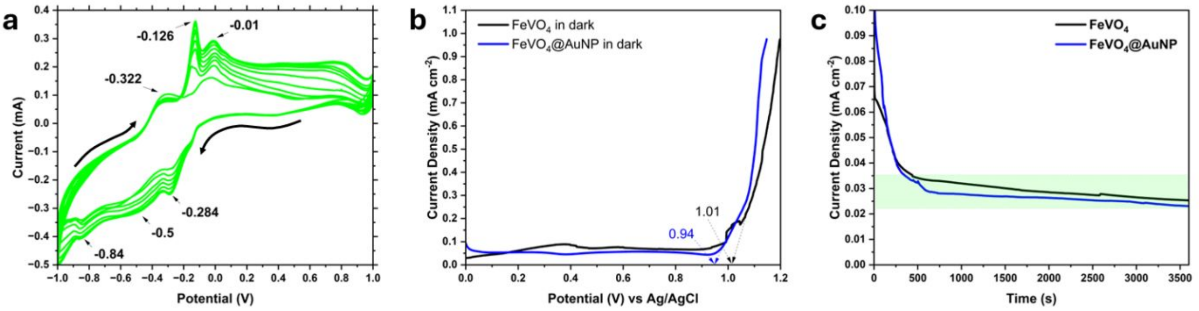

Figure S7. (a) Cyclic voltammogram of $FeVO_4$@AuNP photoelectrode at ambient conditions, scan direction is shown with curved arrows; (b) Linear sweep voltammogram of $FeVO_4$ and $FeVO_4$@AuNP photoelectrodes in dark conditions; (c) Current vs Time chronoamperometry scan of $FeVO_4$ and $FeVO_4$@AuNP under light illumination condition.

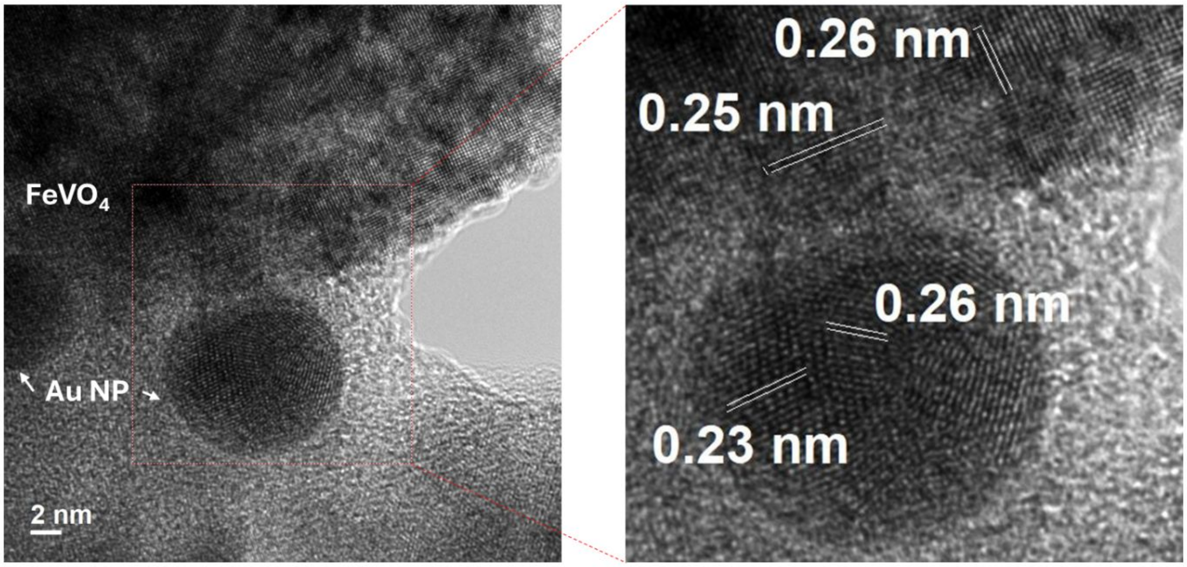

Figure S8. $FeVO_4$@Au interface observed through HRTEM.

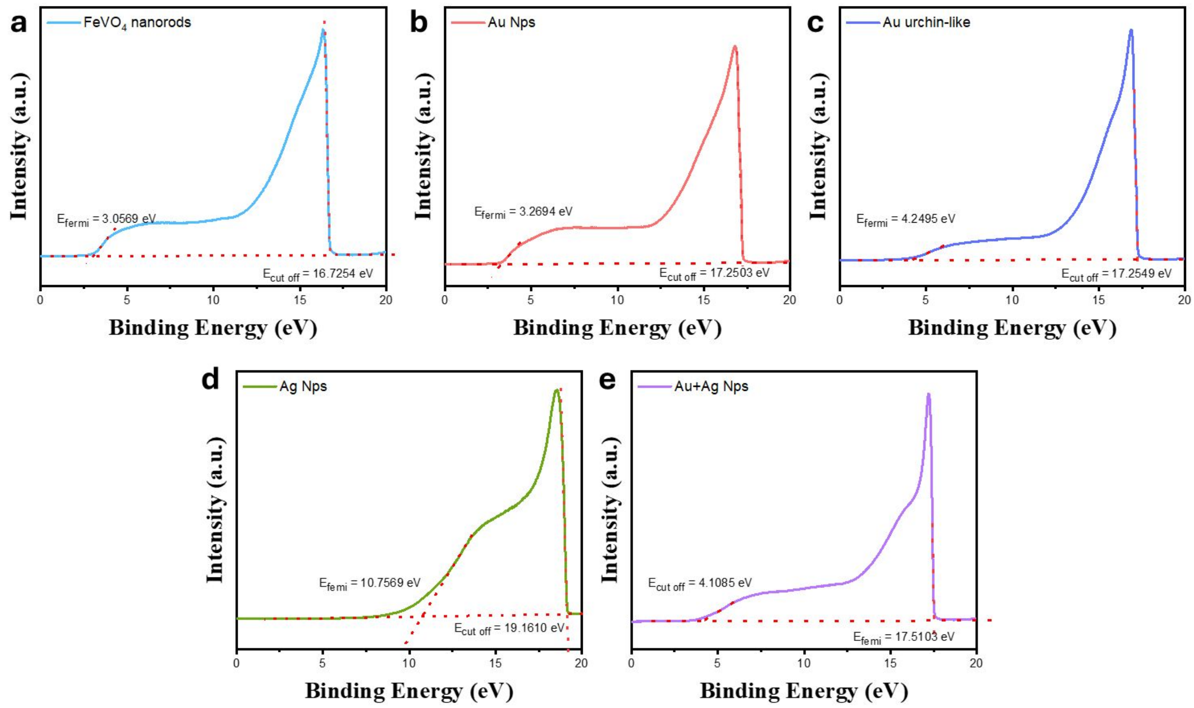

Figure S9. Ultraviolet photoelectron spectra of (a) $FeVO_4$ nanorods; (b) Au; (c) Au-urchin; (d) Ag; (e) Au+Ag.

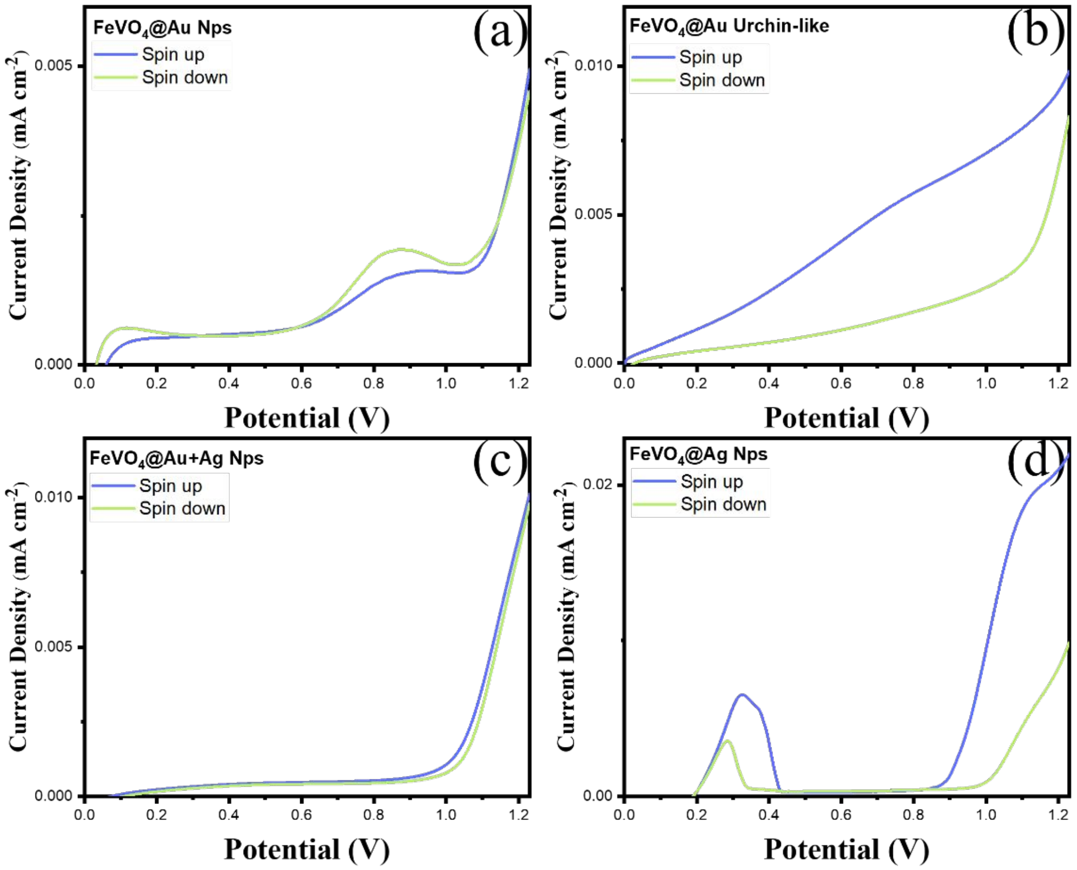

Figure S10. LSV curve of (a) $FeVO_4$@Au NPs under 532 nm laser spin injection, (b) $FeVO_4$@Au urchin-like under 532 nm laser spin injection, (c) $FeVO_4$@Au+Ag NPs under 658 nm laser spin injection, and (d) $FeVO_4$@Ag NPs under 405 nm laser spin injection.

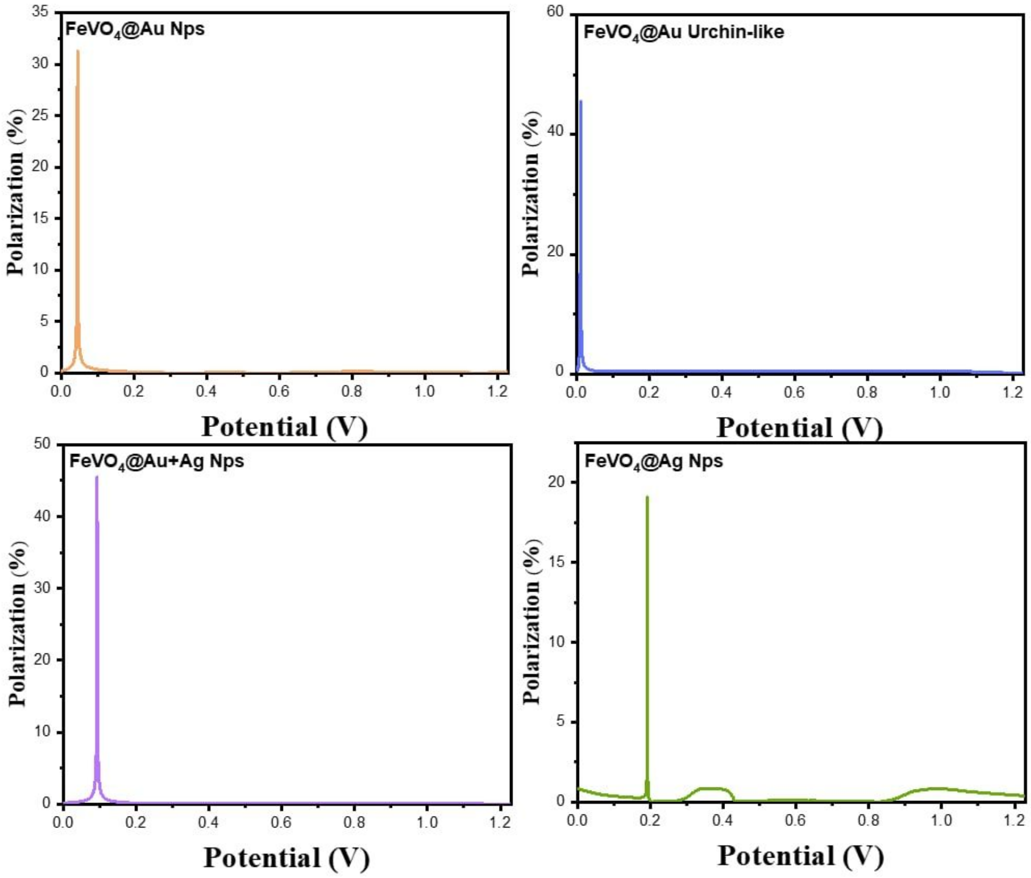

Figure S11. Polarization of (a) $FeVO_4$@Au NPs under 532 nm laser spin injection, (b) $FeVO_4$@Au urchin-like under 658 nm laser spin injection, (c) $FeVO_4$@Au+Ag NPs under 532 nm laser spin injection, and (d) $FeVO_4$@Ag NPs under 405 nm laser spin injection.

Table S2: Hydrogen generation rate of the photoelectrodes.

Photoelectrodes	Hydrogen generation ($\times 10^{-5}$) (mmol s^{-1} cm^{-2})		
		Spin↑	Spin↓
$FeVO_4$	0.00135	-	-
$FeVO_4$@AuNP	0.00207	0.7	0.93
$FeVO_4$@Au-urchin	0.00043	2.47	0.81
$FeVO_4$@AgNP	0.00136	3.36	1.83
$FeVO_4$@Au+Ag	0.00191	2.66	2.55

Table S3: Factors used for GRL predictions

Average Size	Absorbance (nm)	Work function (φ)	E_{BG}	ABPE%	Potential (V)
70	685	4.47	1.81	0.143	0.874
32.7	553	3.95	2.24	0.272	0.788
12	412	2.04	3	0.166	0.822
38.5	522	3.69	2.38	0.256	0.777
88.7	669	3.95	1.85	0.073	0.661
73.5	498	3.75	2.1	0.215621	0.677612
12.4	652	2.68	3.21	0.154237	0.821349
85.7	412	4.15	1.92	0.240031	0.692006
9.3	578	2.45	2.85	0.113096	0.831067
47.8	399	3.3	2.4	0.226266	0.692504
62.1	621	3.98	1.8	0.119346	0.701809
29.6	453	3.1	2.75	0.229797	0.783673
91.2	537	4.25	1.65	0.161069	0.691604
14.8	384	2.55	3	0.226109	0.806216
55.3	690	3.85	2.15	0.081101	0.669044
68.7	479	3.55	2	0.179142	0.673673
36.2	605	2.95	2.5	0.122502	0.766893
81.9	520	4	1.7	0.143045	0.688625
23.5	373	2.8	3.1	0.230178	0.783595
49.1	587	3.4	2.3	0.134292	0.692499
97.8	468	4.35	1.55	0.223783	0.711767
6.7	655	2.35	3.25	0.116017	0.832325
88.3	431	4.1	1.85	0.235019	0.688024
17.2	590	2.65	3.05	0.160997	0.807733
53.9	408	3.45	2.2	0.232279	0.687409
30.5	679	3	2.65	0.118389	0.778796
79.4	489	3.9	1.75	0.191886	0.690262
40.7	569	3.2	2.45	0.146629	0.759341
95.1	445	4.3	1.6	0.252241	0.714492
11.6	374	2.5	3.15	0.226187	0.809076
64.8	614	3.85	1.95	0.091854	0.672472
34.1	456	3.05	2.55	0.221053	0.779705
72.9	531	3.7	2.05	0.175941	0.672283
27.4	598	2.9	2.7	0.130203	0.780897

86.5	417	4.18	1.88	0.244588	0.696367
5.3	643	2.28	3.3	0.117811	0.833717
46.6	477	3.25	2.35	0.20318	0.700882
60.2	608	3.75	2	0.094791	0.671971
92.7	439	4.22	1.62	0.254085	0.713077
21.8	560	2.85	2.95	0.204276	0.796884
38.3	392	3.15	2.6	0.230971	0.772993
75.6	527	3.8	2.08	0.205034	0.676073
19.9	645	2.75	2.98	0.128307	0.800588
50.8	570	3.35	2.25	0.128665	0.67682
84.2	405	4.12	1.9	0.245252	0.696988
16.1	481	2.6	3.12	0.223649	0.806717
55	657	3.55	2.18	0.076755	0.666991
28.7	530	2.95	2.68	0.183415	0.782107
71.4	399	3.65	2.1	0.22604	0.680538
33.9	611	3.02	2.58	0.126834	0.769253
89	423	4.16	1.84	0.24018	0.692385
7.8	675	2.4	3.22	0.113914	0.831526
44.5	586	3.18	2.38	0.127756	0.741659
66.3	468	3.78	2.02	0.234454	0.691751
98.4	452	4.4	1.5	0.239517	0.720697
15.7	558	2.58	3.08	0.182061	0.81274
39.6	389	3.12	2.52	0.230017	0.760129
76.1	520	3.85	2.06	0.211547	0.677353
22.9	672	2.82	2.92	0.12209	0.79387
48.7	493	3.28	2.28	0.178866	0.683031
93.5	435	4.28	1.58	0.259853	0.720114
13.2	397	2.55	3.18	0.226879	0.804552
61.9	619	3.88	1.98	0.100816	0.67751
35.4	541	3.08	2.54	0.165713	0.771064
83.7	408	4.08	1.86	0.247993	0.699887
18.5	584	2.72	3	0.168621	0.804451
52.5	579	3.38	2.22	0.123403	0.671508
96.3	449	4.32	1.57	0.246726	0.715302
10.9	690	2.48	3.14	0.112984	0.828029
63.5	464	3.82	1.92	0.253856	0.715435
37.7	610	3.11	2.56	0.132422	0.76475

74.2	512	3.75	2.12	0.212715	0.676895
20.2	543	2.78	2.96	0.20492	0.800874
58.9	422	3.68	2.04	0.255044	0.709702
91.7	440	4.2	1.63	0.25392	0.712693
8.6	675	2.42	3.2	0.113969	0.830493
41.2	598	3.22	2.42	0.130996	0.756322
77.9	405	3.88	2.07	0.22794	0.681615
26.8	650	2.87	2.8	0.123219	0.7834
45.3	495	3.27	2.37	0.19962	0.71555
85	390	4.14	1.87	0.251248	0.703033
31.5	587	2.97	2.66	0.138948	0.772839
67.6	426	3.72	2.01	0.237357	0.691768
99.1	439	4.38	1.52	0.249855	0.719398
17.4	566	2.62	3.06	0.179589	0.807556
56.8	678	3.6	2.16	0.073826	0.665081
32.1	533	3	2.62	0.176528	0.776452
80.5	413	3.95	1.72	0.258171	0.715034
24.7	581	2.89	2.88	0.179768	0.788786
51.4	573	3.36	2.26	0.12956	0.67697
88.9	430	4.18	1.83	0.242117	0.694304
3.5	689	2.32	3.28	0.111788	0.836423
43	478	3.23	2.39	0.208823	0.731097
65.1	461	3.76	2.03	0.236781	0.693208
94.8	447	4.26	1.59	0.247276	0.712628

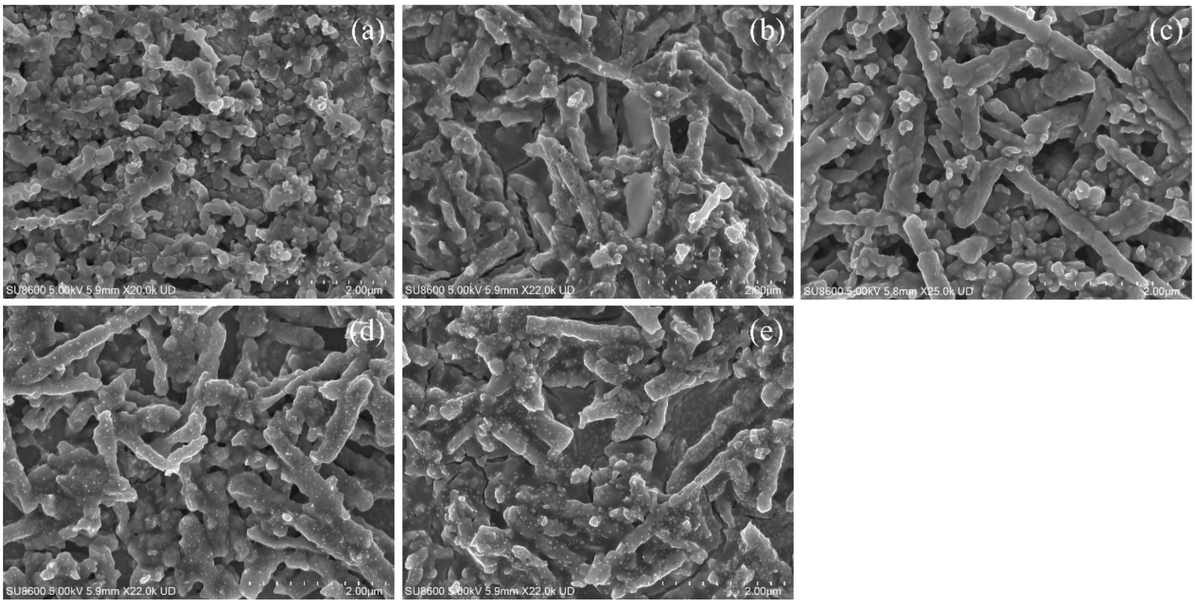

Figure S12. SEM images after the HER reaction of (a) $FeVO_4$ nanorods, (b)$FeVO_4$ nanorods@Au ,(c) $FeVO_4$ nanorods@Au Urchin-like , (d) $FeVO_4$ nanorods@Ag ,(e) $FeVO_4$ nanorods@Au+Ag.

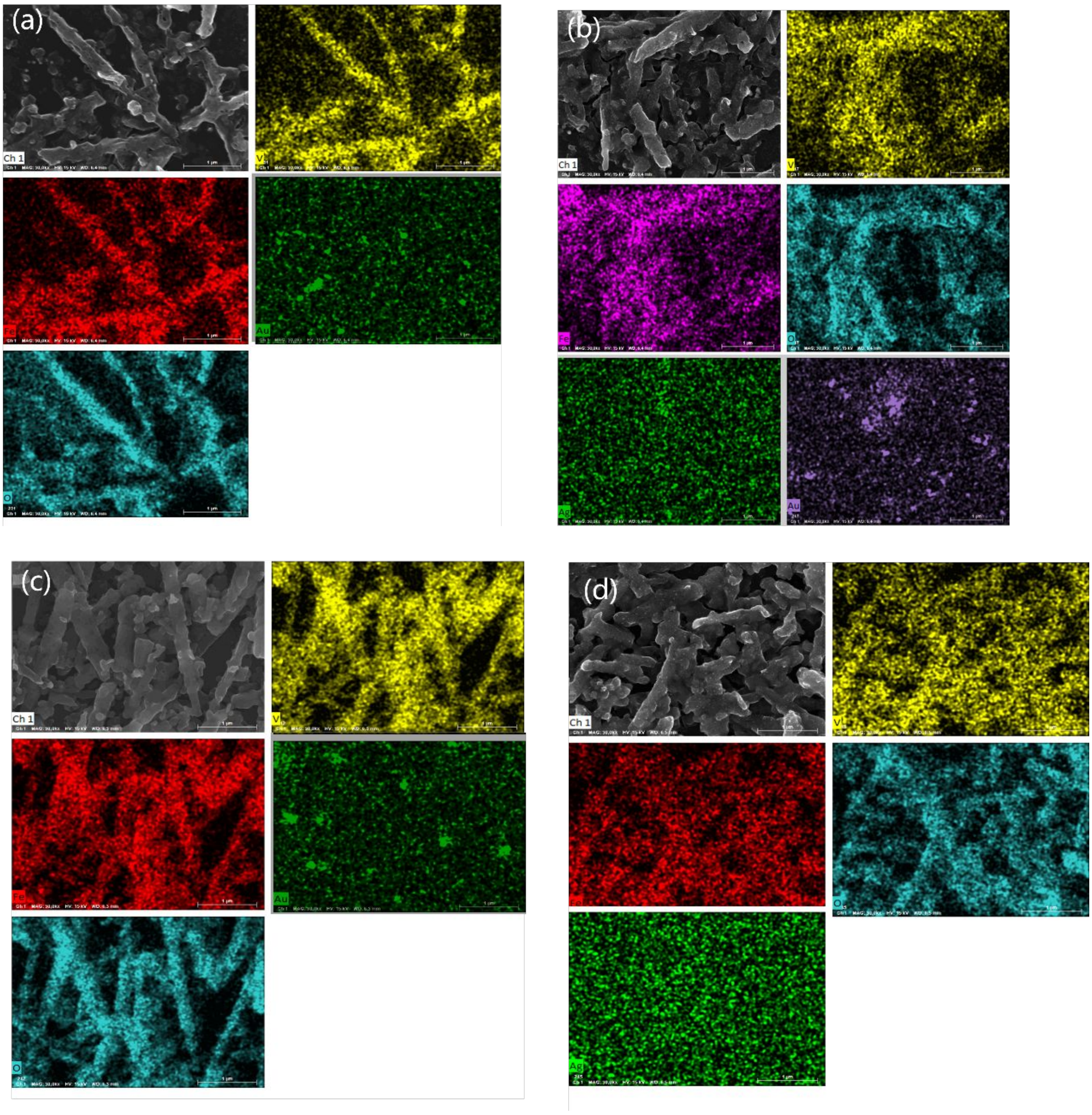

Figure S13. SEM mapping after the HER reaction of $FeVO_4$@ (a)Au NPs, (b) Au+Ag NPs, (c) Au urchin-like, and (d) Ag NPs.

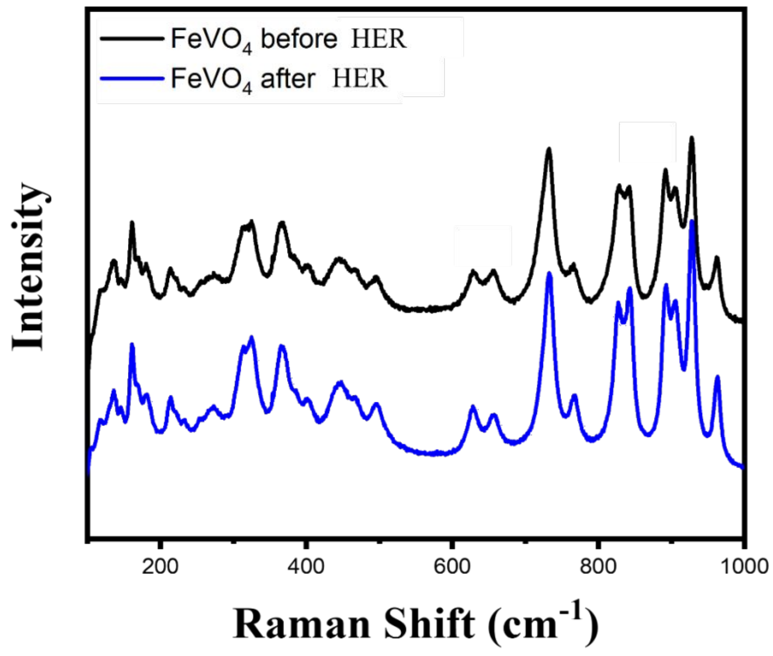

Figure S14. Raman spectra before and after the HER reaction of $FeVO_4$.

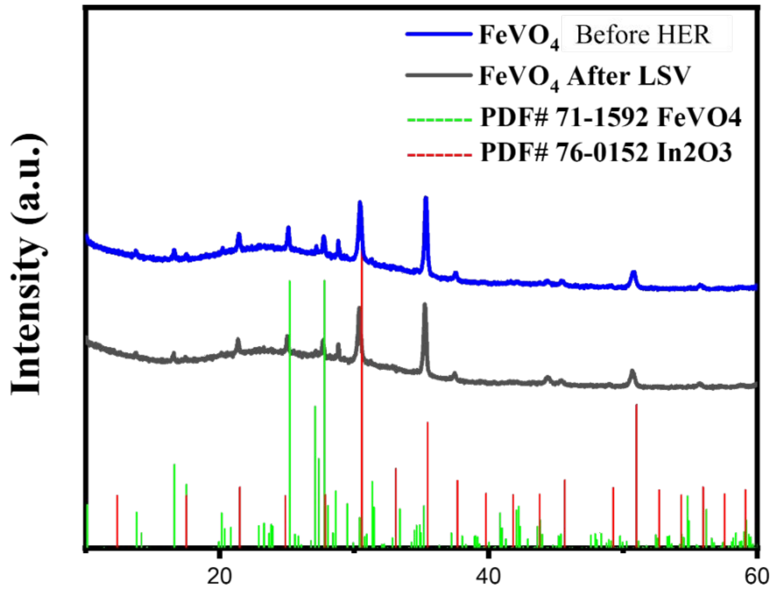

Figure S15. XRD pattern before and after the HER reaction of $FeVO_4$.

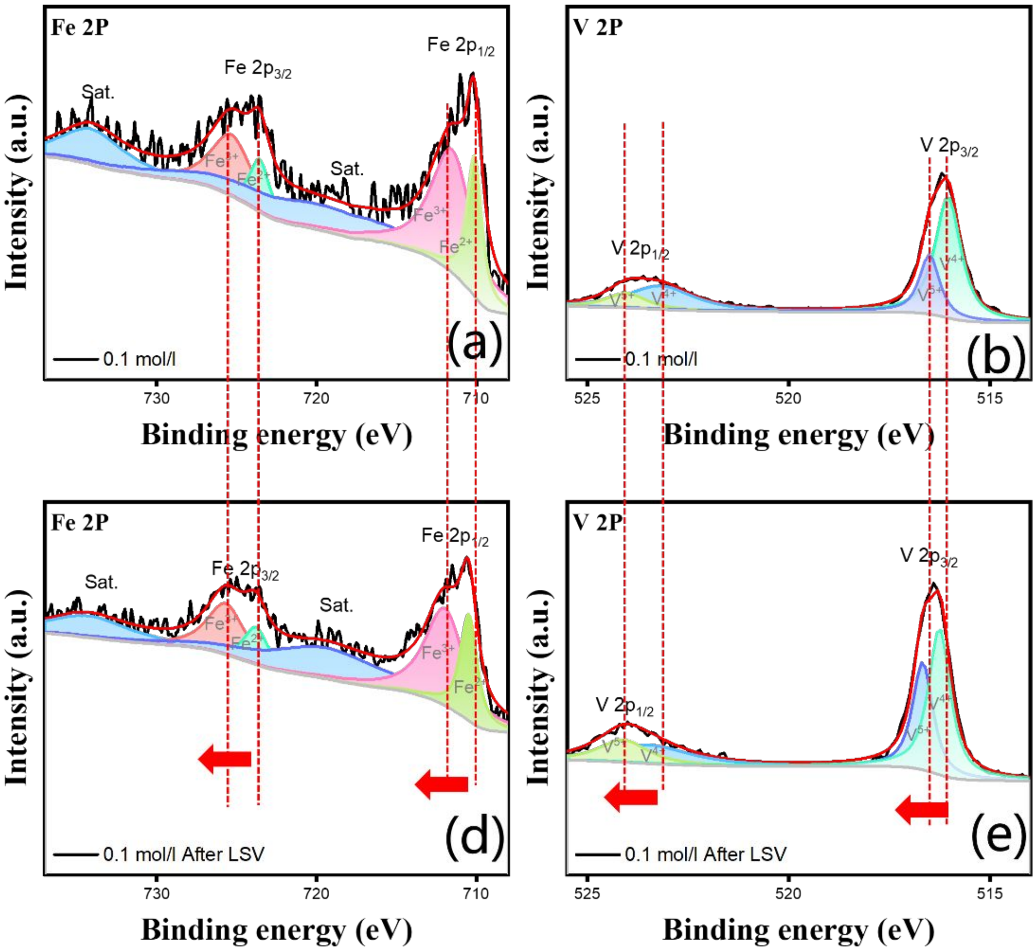

Figure. S16. XPS spectra (a,b) before and (d,e) after the HER reaction of $FeVO_4$.

References

(1) Ravishankar, S.; Ravishankar, S.; Bisquert, J.; Bisquert, J.; Kirchartz, T.; Kirchartz, T. Interpretation of Mott–Schottky plots of photoanodes for water splitting. *Chem Sci* **2022/05/04**, *13* (17). DOI: 10.1039/D1SC06401K.

(2) Sivula, K. Mott–Schottky Analysis of Photoelectrodes: Sanity Checks Are Needed. *Acs Energy Lett* **July 9, 2021**, *6* (7). DOI: 10.1021/acsenergylett.1c01245.
